# Combined GWAS and Transcriptome Analyses Provide New Insights Into the Response Mechanisms of Sunflower Against Drought Stress

**DOI:** 10.3389/fpls.2022.847435

**Published:** 2022-05-03

**Authors:** Yang Wu, Huimin Shi, Haifeng Yu, Yu Ma, Haibo Hu, Zhigang Han, Yonghu Zhang, Zilong Zhen, Liuxi Yi, Jianhua Hou

**Affiliations:** ^1^College of Agricultural, Inner Mongolia Agricultural University, Hohhot, China; ^2^Institute of Crop Breeding and Cultivation, Inner Mongolia Academy of Agricultural and Husbandry Sciences, Hohhot, China

**Keywords:** sunflower, drought stress, genome-wide association studies (GWAS), RNA-seq, single-nucleotide polymorphisms (SNPs), specific-locus amplified fragment sequencing (SLAF-seq)

## Abstract

Sunflower is one of the most important oil crops in the world, and drought stress can severely limit its production and quality. To understand the underlying mechanism of drought tolerance, and identify candidate genes for drought tolerance breeding, we conducted a combined genome-wide association studies (GWAS) and RNA-seq analysis. A total of 226 sunflower inbred lines were collected from different regions of China and other countries. Eight phenotypic traits were evaluated under control and drought stress conditions. Genotyping was performed using a Specific-Locus Amplified Fragment Sequencing (SLAF-seq) approach. A total of 934.08 M paired-end reads were generated, with an average Q30 of 91.97%. Based on the 243,291 polymorphic SLAF tags, a total of 94,162 high-quality SNPs were identified. Subsequent analysis of linkage disequilibrium (LD) and population structure in the 226 accessions was carried out based on the 94,162 high-quality SNPs. The average LD decay across the genome was 20 kb. Admixture analysis indicated that the entire population most likely originated from 11 ancestors. GWAS was performed using three methods (MLM, FarmCPU, and BLINK) simultaneously. A total of 80 SNPs showed significant associations with the 8 traits (*p* < 1.062 × 10^−6^). Next, a total of 118 candidate genes were found. To obtain more reliable candidate genes, RNA-seq analysis was subsequently performed. An inbred line with the highest drought tolerance was selected according to phenotypic traits. RNA was extracted from leaves at 0, 7, and 14 days of drought treatment. A total of 18,922 differentially expressed genes were obtained. Gene ontology and Kyoto Encyclopedia of Genes and Genomes analysis showed up-regulated genes were mainly enriched in the branched-chain amino acid catabolic process, while the down-regulated genes were mainly enriched in the photosynthesis-related process. Six DEGs were randomly selected from all DEGs for validation; these genes showed similar patterns in RNA-seq and RT-qPCR analysis, with a correlation coefficient of 0.8167. Through the integration of the genome-wide association study and the RNA-sequencing, 14 candidate genes were identified. Four of them (LOC110885273, LOC110872899, LOC110891369, LOC110920644) were abscisic acid related protein kinases and transcription factors. These genes may play an important role in sunflower drought response and will be used for further study. Our findings provide new insights into the response mechanisms of sunflowers against drought stress and contribute to further genetic breeding.

## Introduction

Sunflower (*Helianthus annuus. L*) belongs to the Compositae family (Schilling and Heiser, 1981), and is native to North America (Schilling and Heiser, 1981). As one of the major oilseed crops in the world, sunflower is considered an important source of high-quality oil and dietary fiber for human health (Khan et al., 2015). The world harvested area of sunflower seed has increased by 20% (from 23.07 million hectares to 27.87 million hectares), and the production has increased by more than 50% (from 31.45 million tons to 50.23 million tons) from 2010 to 2020 (FAO, 2021). China is the sixth-largest sunflower-producing country in the world. The main production areas of sunflowers in China are in the northwest region, such as Inner Mongolia Autonomous Region and Xinjiang Uygur autonomous region. The sunflower is an important economic source for local farmers, and the status of sunflower production directly affects farmers' living standards.

The global average temperature has risen by about 0.85°C from the year 1880 to 2012 (Adopted, 2014), resulting in a series of extreme weather events, such as heavy rains, flooding, drought, and desertification. Among them, drought is the most serious abiotic stress limiting global agricultural production (Wilhite and Buchanan-Smith, 2005). A persistent drought can cause a large number of deaths and force large-scale migration, while severe droughts can even impact human civilization (Ault, 2020). With the continued climate change and population growth, drought may pose a serious threat to global and regional food security in the coming decades (Riddell et al., 2018). Due to the strong root system, the sunflower was considered to be relatively tolerant to water stress. They are often seeded on beds and ridges with poor moisture conditions where many other crops are unable to survive (Hussain et al., 2018). As a result, it is more susceptible to drought stress leading to yield reduction (Pasda and Diepenbrock, 1990; Adeleke and Babalola, 2020; Grasso et al., 2020). Studies have shown that drought stress in sunflower seedlings can lead to severe yield loss (Mwale et al., 2003; Rauf and Ahmad Sadaqat, 2008).

The sunflower drought stress response behavior involves a series of changes in morphological, physiological, and molecular levels. The drought stress negatively influenced seed germination and seedling emergence at the germination stage (Kaya et al., 2006). Drought stress at the vegetative stage reduces plant height (PH), leaf surface area (LSA), and biomass production while causing pollen sterility at the reproductive stage (Turhan and Baser, 2004; Hussain et al., 2008). From a physiological perspective, drought affects the uptake of water and nutrition, leads to a reduction of relative water content (RWC), and the turgor of cells (Hussain et al., 2008, 2016; Ibrahim et al., 2016). Plants respond to drought stress by reducing water evaporation through stomatal closure. As a result, it also reduces the photosynthetic rate (Flexas et al., 2004). The decreased photosynthesis rate leads to a decrease in CO_2_ fixation, which affects the regeneration of the final acceptor of the electron transport chain (NADP^+^). The leaked electrons flow to O_2_ to produce reactive oxygen species (ROS) (Flexas et al., 2004). ROS cause oxidation of membrane lipids, resulting in decreased cell membrane stability. The decrease in cell membrane permeability results in the accumulation of the relative electrical conductivity (REC) and malondialdehyde (MDA) (Gunes et al., 2008). From the molecular level, plants involve a series of pathways for signal perception, transduction, gene expression, and other stress metabolites to accommodate drought. Drought-induced genes can mainly be classified into two groups. The first group constitutes genes whose products directly function in tolerance to stress, such as LEA proteins, osmolytes, proline (Pro), CAT, POD. Another group includes genes playing a role in signal transduction as well as the regulation of gene expression including various transcription factors (TF), protein kinases (PK), and transcriptional regulators (TR) (Lata et al., 2015).

Some agronomic measures can mitigate the damage of drought impact on plants, such as exogenous applications of plant hormones, osmotic regulators, and mineral nutrients (Salami and Saadat, 2013; Rabert et al., 2014). However, these changes are not heritable, and need additional labor, capital, and technology investment. Coping with drought through the breeding approach is usually the most effective and economical strategy. The genetic modification within the plant is heritable. Once a gene is introduced into a breeding material, it will be a permanent source of drought tolerance (Rauf, 2008). Drought tolerance in plants is a complex quantitative trait involving many micro-effective genes (Blum, 2011). Molecular-based plant drought resistance breeding is a hot spot in recent years (Wang and Qin, 2017). Previous studies on the molecular mechanism of sunflower drought resistance were mostly based on linkage analysis (Kiani et al., 2007; Poormohammad Kiani et al., 2009; Haddadi et al., 2011). However, the linkage analysis population was on two parents with significantly different phenotypes and the recombinant inbred lines (RILs). Only genes in RILs that show a significant difference between parental lines could be detected.

Genome wide association study (GWAS) is an observational study to detect associations between genetic variants and traits in individuals (Togninalli et al., 2018). Compared to linkage analysis, GWAS uses a natural population, which eliminates the need to construct a population. Therefore, the time consumption is greatly reduced. The use of natural populations allows GWAS to simultaneously detect many natural allelic variations (Ma et al., 2018). In addition, the natural population contains all the historical recombination information and thus provide relatively higher detection accuracy than bi-parental populations (Kofsky et al., 2020). GWAS has been widely used in plant drought research, such as wheat (*Triticum aestivum L*.), cotton (*Gossypium herbaceum L*.), rice (*Oryza sativa L*.), and potato (*Solanum tuberosum L*.) (Ma et al., 2016; Mwadzingeni et al., 2017; Hou et al., 2018; Tagliotti et al., 2021). RNA-sequencing (RNA-Seq) is another attractive omics tool to identify differentially expressed genes (DEGs) under different conditions. Further analysis can provide insight into the changes in the DEGs expression level, important biological processes, and pathways (Zhang et al., 2017). Combining GWAS with RNA-seq can decrease the higher false-positive rate (FDR) inherent in GWAS analysis, and improve the accuracy of gene selection (Xie et al., 2019; Wang et al., 2022). However, to our knowledge, there are no relevant studies on sunflowers.

Molecular marker-based genotyping is an important step in GWAS analysis. Most traditional molecular markers were based on sequence length polymorphism. However, it could not be used for large-scale genotyping due to low throughput (Sun et al., 2013b). Whole gene sequencing technology is restricted in its use for non-model organisms due to population size and price (Muir et al., 2016). One strategy to reduce the sequencing cost was to reduce representation libraries (RRL). Specific length amplified fragment sequencing (SLAF) is one of the representative techniques, which uses specific enzymes to digest the genomes, and select a given size range of restriction fragments based on personalized research purposes (Sun et al., 2013b). This approach maintains the marker density while reducing the volume of sequencing, lowering the cost.

In this study, we performed a GWAS analysis of 226 sunflower varieties based on SLAF-seq. Then, a drought-tolerant accession was selected for RNA-seq analysis. Several important candidate genes were obtained using a combined analysis. Our research objectives were to (1) investigate the phenotypic variations among accessions under different water conditions; (2) develop new drought-related SNPs and identify genetic variants; (3) understand gene expression patterns under different drought stress time points, and reveal important biological processes and pathways; (4) obtain important genes associated with drought tolerance.

## Materials and Methods

### Plant Materials and Growth Condition

A total of 226 sunflower inbred lines were collected from different countries (Australia, U.S.A., and France) and different provinces in China (Inner Mongolia, Ningxia, Xinjiang, Liaoning, Jilin). Seventy-three of them were provided by the Inner Mongolia Academy of Agriculture and Animal Husbandry, and 153 were kept in our laboratory. The experiment was conducted in the summer of 2019 at the Inner Mongolia Agricultural University, China (111.71, 40.82, 1,000 m above sea level). Seeds with fully mature, healthy, and uniform sizes were sorted for drought-stress experiments. After sterilization with 0.2% (w/v) mercuric chloride (HgCl_2_), all seeds were rinsed several times with distilled water and soaked in deionized water for 24 h. Then the seeds were sown in plastic flowerpots (25 × 19 × 16 cm) filled with 3 kg soil (sandy soil and organic humus in a ratio of 2:1). Each pot was planted with 10 seeds and each accession had 6 pots. To avoid interference from natural rainfall and other factors, all pots were placed in a greenhouse (light/dark cycles: 14 h/10 h; 28/22°C; 45 ± 5% relative humidity) without water and nutritional limitation.

### Experimental Design and Drought Treatments

When seedlings grew to the stage of three leaves, six pots of each accession were randomly and equally divided into two groups. Each group contained three pots as three biological replicates. The different watering regime was imposed on these two groups. One group continued to irrigate with sufficient water, and maintain the soil moisture content of 30 ± 2% as a control group (WW). Another group kept the soil moisture content to 10 ± 2% as a treatment group (DS). The soil moisture content of each pot was determined at 9 a.m. every day using the weight method described by Soni and Abdin (2017) and supplemented with water according to the target soil moisture content.

### Phenotypic Evaluation and Statistical Analysis

The experiment lasted for 15 days, then 5 plants were randomly selected from each pot for phenotypic evaluation. Plant height (PH) was measured directly with a ruler. Leaf surface area (LSA) was calculated by the leaf area co-efficient method (Alza and Fernandez-Martinez, 1997). Root shoot ratio (RSR) was measured by the gravimetric method. Total root length (RL), root volume (RV), and root surface area (RSA) were measured with an LA-S root scanner (Wanshen Testing Technology Co., Ltd., Hangzhou, China). The relative water content (RWC) was detected using the saturate water method by Galmes et al. (2011). The chlorophyll concentration was assessed using a SPAD chlorophyll meter (TYS-A, TOP Instrument Co., Ltd., Hangzhou, China).

Data were analyzed using SPSS software (SPSS for Windows, V20.0.0, SPSS, Chicago, Illinois). Normality distribution was preliminarily assessed by a one-sample Kolmogorov-Smirnov's goodness-to-fit test (K-S test). For statistical differences between WW and DS growth condition, the Student *t*-test (normal distribution) and Wilcoxon signed-rank test (non-normal distribution) was used. Spearman non-parametric correlations were used to determine the correlation coefficient and statistical significance. Corrplot and Pheatmap R package were used to visualize the correlation.

### Genomic DNA Extraction and Restriction Enzyme Selection

Total genomic DNA was extracted from 100 mg of fresh leaves by the CTAB method with a plant genomic DNA kit DP305 (Tiangen Biotech, China). To ensure it met the requirements for SLAF-seq (concentration ≥ 20 ng/μl; volume ≥ 30/μl), the concentration and quality of DNA were determined using a Nanodrop 2000 spectrophotometer (Thermo Scientific, Waltham, MA, USA).

The SLAF-seq technique requires breaking the genome into small fragments using restriction enzymes. Then selecting restriction fragments of a specific length range (defined as SLAF-seq) for sequencing. To evaluate the number of target fragments produced *via* different combinations of restriction enzymes, a *in silicon* pre-experiment for enzyme selection was conducted. The criteria for enzyme selection were as follows: (1) the proportion of restriction fragments located in repetitive sequences is as low as possible; (2) The restriction fragments are distributed evenly on the genome as far as possible; (3) Consistency between the length of restriction fragments and the specific experimental system (Davey et al., 2013); (4) The number of restriction fragments with lengths 364–464 pb (SLAF tags in sunflower) should exceed 300,000.

### SLAF Library Construction and High Throughput Sequencing

The SLAF library construction and high-throughput sequencing were performed as described by Sun et al. (2013b). After a series of polymerase chain reactions (PCR), adapter ligation reactions, and agarose gel purification, the SLAF-tags were isolated and subjected to PCR amplification following the guide of Illumine sample preparation. The paired-end sequencing was performed on an Illumina HiSeq 2500 platform (Illumina Inc., San Diego, CA, USA) at Beijing Biomarker Technologies Corporation (Beijing, China). Sequencing quality was estimated by measuring the guanine-cytosine (GC) content and Q30 ratio. A *Q* value of 30 represents a 0.1% error probability and 99.9% confidence level. Reads with >90% identity were clustered into a single SLAF-tag using BLAT software, and SLAF-tags with a sequence that varied across samples were defined as polymorphic SLAF tags (Zhang et al., 2018). To test the accuracy of the restriction enzyme digestion protocol, we used the genome of *Oryza sativa ssp. japonica* as a control (374.30 Mb, http://rapdb.dna.affrc.go.jp/).

### SNP Genotyping and Linkage Disequilibrium Analysis

All reads were processed for quality control and filtered using Seqtk (https://github.com/lh3/seqtk) software. High-quality paired-end reads were aligned to the reference genome (https://ftp.ncbi.nlm.nih.gov/genomes/all/annotation_releases/4232/100/GCF_002127325.1_HanXRQr1.0/) using Burrows-Wheeler Aligner (BWA) software (Li and Durbin, 2009). SNP calling was conducted using the HaplotypeCaller function of Genome Analysis Toolkit (GATK) (McKenna et al., 2010). The VCF files obtained by GATK were converted to PLINK files using VCFtools (v0.1.16) (Danecek et al., 2011). SNPs with an integrity ratio of <0.8 and MAF <0.05 were filtered out *via* PLINK software (v1.90b6.21) (Purcell et al., 2007). Linkage disequilibrium (LD) was estimated by measuring the squared allele frequency correlations (*r*^2^) (VanLiere and Rosenberg, 2008) between pairs of SNPs *via* PLINK software, with *r*^2^ = 1 indicating complete LD, and *r*^2^ = 0 indicating absent LD. LD decay extent was defined as the physical genomic distance at which the *r*^2^ decreased to half of its maximum value. PopLDdecay software (Zhang et al., 2019) was used to visualize the mean *r*^2^ of all chromosomes within the 100 kb region.

### Population Structure Analysis

Based on the filtered SNPs, population analysis, phylogeny analysis, and principal component analysis (PCA) were performed in turns. Admixture software v1.3.0 (Alexander et al., 2009) was used to analyze the population structure. The number of underlying population groups *K* was predefined as 1–13 using the maximum likelihood estimation approach. The cross-validation errors (CV) for each *K* value were calculated. The *K* value with the lowest CV error was selected as the optimal number of populations. The Pophelper R package was used to make multiline plots (Francis, 2017). The genetic distances were calculated using VCF2Dis-1.45 (https://github.com/BGI-shenzhen/VCF2Dis). The FastME (v 2.0) software (Lefort et al., 2015) was used to convert the mat file obtained in the previous step into a distance matrix file (^*^nwk). The phylogenetic trees were constructed using the neighbor-joining method in the iTOL server (https://itol.embl.de/) (Letunic and Bork, 2021). PCA was performed using PLINK software by the –pca function. The first three components were used to plot the PCA *via* the rgl (v. 0.107.14) R package (Adler et al., 2003).

### Genomic-Wide Association Study

The GWAS analysis was conducted using three methods: mixed linear model (MLM), Fixed and random model Circulating Probability Unificatin (FarmCPU), and Bayesian-information and Linkage-disequilibrium Iteratively Nested Keyway (BLINK) in GAPIT R package (Lipka et al., 2012). The phenotypic data of each accession was represented using two indices: stress tolerance index (STI) (Fernandez, 1992), and stress susceptibility index (SSI) (Fischer and Maurer, 1978). These were calculated as follows:


$$\begin{array}{l} STI=\frac{Y_{si}\times Y_{pi}}{{{\bar{Y}}_{pi}}^{2}} \end{array}$$

$$\begin{array}{l} SSI=\frac{1-\frac{Y_{si}}{Y_{pi}}}{1-\frac{{\bar{Y}}_{si}}{{\bar{Y}}_{pi}}} \end{array}$$

where *Y*_*si*_ = performance of a genotype under stress; *Y*_*pi*_ = performance of the same genotype under control conditions; ${\bar{Y}}_{\text{si}}=$ mean Y_si_ of all genotypes, ${\bar{Y}}_{\text{pi}}$ = mean Y_pi_ of all genotypes.

The first three principal components were used as covariates. The GAPIT uses genotype data to automatically generate kinship matrix and calculate population structure according to the needs of different methods. For the identification of true marker-trait association, the significant *p*-value was set as *p* < 1.062 × 10^−6^ (*p* = 0.1/*n*; *n* = total markers used, which is roughly a Bonferroni correction, corresponding to −log_10_(*p*) = 5.97, blue line in the Manhattan plots) (Zhou et al., 2017). The Manhattan plot was used to show the correlation between SNP and phenotypic traits. The Quantile-quantile (Q-Q) plot was used to display the level of difference between observed and predicted values. Both the Manhattan plots and Q-Q plots were constructed using CMplot R package (Yin, 2018).

### GWAS Candidate Gene Search and Combined Analysis

The region of GWAS candidate genes was defined by the average LD decay distance. Genes located within 20 kb flanking regions on either side of the significantly associated SNPs were considered as candidate genes. Function annotations were conducted using the Eggnog (Huerta-Cepas et al., 2019) and Pfam (Bateman et al., 2004) software. The blast software was used to search for *Arabidopsis thaliana* genes homologous to candidate genes in the TAIR database (https://www.arabidopsis.org). Transcription factors (TF), protein kinase (PK), and transcriptional regulators (TR) were identified using iTAK software (Zheng et al., 2016).

### Material Screening and RNA-Sequencing

To reveal important biological processes and significant pathways involved in sunflower drought-response, and narrow down the candidate genes, RNA-seq was conducted. We screened the 226 GWAS accessions based on phenotypic evaluation results. A comprehensive drought tolerance coefficient value (*D*-value) was used to evaluate the drought tolerance of all accessions (Li et al., 2015). The *D*-value integrated the results of multi-traits measured under two watering regimes and can represent the comprehensive drought tolerance of an accession. Finally, an inbred line with the highest *D*-value was selected and named “K58” (Zilong et al., 2021).

The drought stress experiment was the same as GWAS. Young leaves were sampled at 0, 7, and 14 days after drought treatment. Total mRNA was isolated using the RNA prep pure plant kit DP411 (Tiangen Biotech, China) according to the instruction manual. A total of 1 μg RNA per sample was used for cDNA library construction. Sequencing libraries were generated using NEBNext UltraTM RNA Library Prep Kit for Illumina (NEB, USA) following the manufacturer's recommendations. The quality of libraries was assessed through the Agilent Bioanalyzer 2100 system. After the quality test, all samples were sequenced in the Illumina Novaseq 6000 system, and 150-bp paired-end sequences were obtained (raw reads). Clean reads were obtained by eliminating reads containing ploy-N, reads containing adapter and low-quality reads from raw reads. The Q30, GC content of clean reads were calculated simultaneously.

### Analysis of Differentially Expressed Genes

Differentially expressed genes analysis was conducted using the HISAT2-Stringtie(merge)-DESeq2 pipeline. High-quality clean reads were aligned to the reference genome using the Hisat2 software (version 2.2.1) (Kim et al., 2015) with default parameters. In the gene count step, we used a “Transcript merge mode” *via* StringTie software (Pertea et al., 2015). Briefly, the alignment files (*.BAM) of each sample was converted to GTF file using StringTie software. Then all the GTF files were merged into one single file containing a non-redundant set of transcripts. This file was then used as a reference to recalculate the count for each gene. With this model, novel genes/transcripts can be identified that differ from the reference genome.

A python script [prepDE.py (https://ccb.jhu.edu/software/stringtie/dl/prepDE.py)] was used to generate a gene count matrix from the GTF file of each sample. Normalization and differential expression analysis were performed using DESeq2 R packages (Love et al., 2014). By default, DESeq2 computes a Benjamini-Hochberg adjusted *p*-value (*P*_*adj*_) to control the false discovery rate (FDR) (Anders and Huber, 2012). The “Fold Changes” of a gene is the FPKM ratio at day 7 (or 14) to that at day 0. For comparison purposes, we take the logarithm of the fold change and calculate the absolute value (|log_2_(Fold Changes)|). The |log2(Fold Changes) | of a gene equal to 1 means that the expression level of this gene has doubled or halved. Genes with *P*_*adj*_ ≤ 0. 01 and |log_2_(Fold Changes) | ≥ 1 was considered as DEG.

### Enrichment Analyses of Gene Ontology and KEGG Pathways

Gene Ontology (GO) and Kyoto Encyclopedia of Genes and Genomes (KEGG) analyses were performed to reveal the biological functions and pathways of DEGs. The sequence file of each gene was input into Eggnog software (version 2.0.1) to obtain gene annotation (Huerta-Cepas et al., 2019). GO and KEGG analysis was conducted using the ClusterProfiler (version 4.0.0) R package (Yu et al., 2012). Only GO-terms or KEGG pathways with *p*-value <0.05 were screened for subsequent analysis. The REVIGO program (http://revigo.irb.hr/) was used to remove redundant GO-terms (Supek et al., 2011).

### RT-qPCR Validation

To validate RNA-seq results, reverse transcription quantitative PCR (RT-qPCR) was conducted on 6 randomly selected DEGs with three technical replicates. Experimental samples are the same as for RNA-seq. Reverse transcription was conducted using Biomarker Script II 1st Strand cDNA Synthesis Kit (Biomarker Technologies, Beijing, China) with Oligo d(T)_23_ VN as a primer, and qPCR reactions were performed with Biomarker 2X SYBR Green Fast qPCR Mix (Biomarker Technologies, Beijing, China) on the FTC-3000 qPCR system (Funglyn Biotech Inc., Toronto, ON, Canada). Gene expression levels were calculated using the method of 2^−ΔΔCt^ according to Livak and Schmittgen (Livak and Schmittgen, 2001), and standard deviation was calculated among three biological replicates. The 18S rRNA gene was used as the endogenous control (Ebrahimi Khaksefidi et al., 2015).

### Combined Analysis of GWAS and RNA-Seq

To reduce the number of candidate genes, we conducted a combined analysis. The two gene sets obtained by GWAS and RNA-seq were subjected to the intersection operation. Genes within the intersection were considered to be important genes and were investigated in depth.

## Results

### Phenotypic Variation Among Accessions

**Figure 1:**
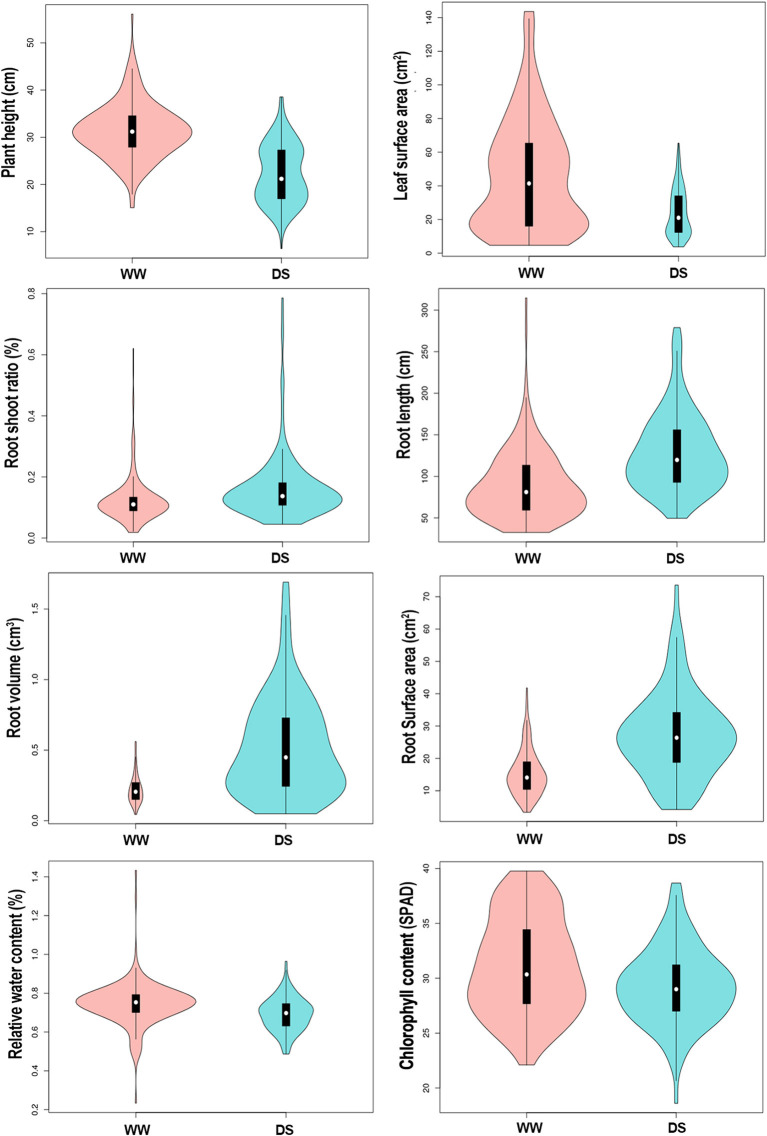
Vioplot visualizing the 8 physiological traits of sunflower in response to different water treatments. *Y*-axis represent the density distribution of all 226 samples. WW, well-water growth condition; DS, drought-stress growth condition.

Drought stress led to different degrees of changes in all phenotypic traits (Figure 1; Table 1). Drought stress inhibited plant height (PH). Mean PH was 31.37 cm (ranged from 15.07 to 56.10 cm) at WW condition, whereas it was 22.23 cm (ranged from 6.4 to 38.55 cm) under DS conditions. Over 90% of the accessions (208/226) had a decrease in PH under drought stress.

**Table 1:** Descriptive statistics values for traits of 226 sunflowers under drought stress.

**Traits**	**Trt**.	**Min**.	**Max**.	**Mean**	**SD**.	**CV. (%)**	**Skewness**	**Kurtosis**
Plant height	WW	15.07	56.10	31.37	5.80	18.48	0.50	1.56
	DS	6.40	38.55	22.23	6.16	27.72	0.25	−0.65
Leaf surface area	WW	4.62	143.62	46.34	33.30	71.85	0.91	0.30
	DS	3.73	65.36	24.21	14.03	57.93	0.71	−0.31
Root shoot ratio	WW	0.02	0.62	0.12	0.08	61.42	3.24	14.85
	DS	0.05	0.79	0.16	0.11	65.49	3.21	13.05
Root length	WW	32.56	314.68	89.47	40.83	45.63	1.63	5.11
	DS	49.66	279.06	128.90	46.76	36.28	0.96	0.86
Root volume	WW	0.04	0.56	0.22	0.11	46.99	1.21	1.53
	DS	0.05	1.69	0.52	0.35	67.17	1.01	0.88
Root surface area	WW	3.33	41.79	15.50	7.04	45.43	1.00	1.03
	DS	4.22	73.59	27.34	13.19	48.23	0.74	0.88
Relative water content	WW	0.23	1.43	0.74	0.11	14.59	0.84	11.26
	DS	0.49	0.96	0.69	0.09	12.54	0.10	0.19
SPAD	WW	22.10	39.77	31.08	4.38	14.09	0.19	−0.97
	DS	18.60	38.67	29.31	3.50	11.93	0.18	0.42

*Trt., Treatment; Min., Minimum; Max., Maximum; SD, Standard deviation; CV, Coefficient of variance; CK, Well water condition; DS, Drought stress condition*.

Mean leaf surface area (LSA) was 46.34 cm^3^ (ranged from 4.62 to 143.62 cm^3^) for the WW condition compared with 24.21 cm^3^ (ranged from 3.73 to 65.36 cm^3^) for the DS condition. Over 88% (200/226) of the accessions had a decrease in LSA under drought stress.

The root-shoot ratio (RSR) increased slightly under the DS condition compared with in WW condition. Mean RSR was 0.16 (ranged from 0.05 to 0.79) under DS condition, whereas it was 0.12 (ranged from 0.02 to 0.62) under WW condition, with 71.7% (162/226) of the accessions showing an increased RSR under DS conditions. Notably, drought stress significantly increased three root-related traits, the average root length (RL), root volume (RV), and root surface area (RSA) increased by 44.1, 131, and 76.4% under DS condition compared with plants under WW condition. Among the 226 accessions, 77.4% (175/226), 83.2% (188/226), 83.2% (188/226) of them showed an increased RL, RV, and RSA under drought conditions, respectively. Drought stress has relatively little effect on the relative water content (RWC) of sunflower leaves, and the mean value was reduced from 0.74% under WW condition to 0.69% under the DS condition, with a reduction rate of 7.3%. Among 226 sunflower plants, 83.6% (189/226) had lower RWC under the DS condition. Similarly, the SPAD value was also decreased slightly in DS compared to WW, with a reduction rate of 5.7%. Mean values were 31.08 (ranged from 22.1 to 39.77) and 29.31 (ranged from 18.6 to 38.67) under WW and DS, respectively, and 72.6% (164/226) accessions showed a decreased SPAD value under DS condition.

The coefficient of variation (CV) was used to describe the variance within accessions. In this study, the CV of some traits was very high, the average CV among all traits were 40.36%, varying from 11.94 to 71.86%. It shows that our experiment materials have strong heterogeneity. RSR had the highest CV values (61.42–65.49%) while the SPAD value showed the lowest CV values (11.94–14.09%) (Supplementary Table 1).

**Figure 2:**
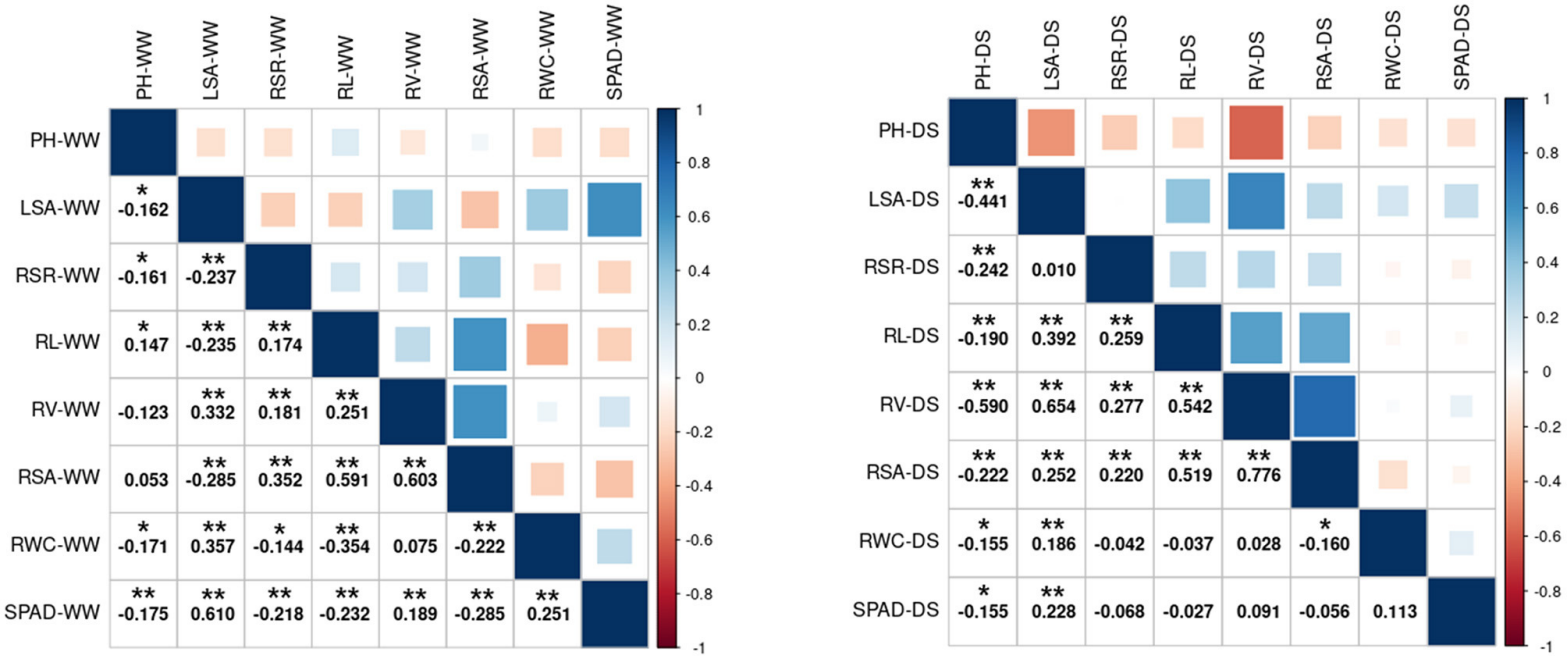
Spearman's correlation analysis between the 8 drought-related traits under two water condition. Left: Under WW growth condition. Right: Under DS growth condition. WW, well-water growth condition; DS, drought-stress growth condition; PH, plant height; LSA, leaf surface area; RSR, Root shoot ratio; RL, Root length; RV, Root volume; RSA, Root surface area; RWC, Relative water content; SPAD, SPAD value. * and **Significant at the 0.05 and 0.01 probability levels between genotypes, respectively.

The correlation between the same indicator under different conditions is shown in Supplementary Figure 1. The correlation coefficients of LSA and SPAD were higher than 0.6 in the WW vs. DS, while the correlation coefficients of RSA, RL, and RSR were all lower than 0.1. The correlation between different indicators under the same condition is shown in Figure 2. The three root-related indexes (RL, RV, and RSA) showed positive correlation under both WW and DS growth conditions. Under DS conditions, RV was positively correlated with RSA (spearman Cor. = 0.776). whereas negatively correlated with PH (spearman Cor. = −0.59). Under WW conditions, LSA is positively correlated with SPAD with a spearman correlation coefficient of 0.61.

### SLAF-Sequencing, Genotyping, and Linkage Disequilibrium

Enzyme digestion efficiency is an important indicator of SLAF-seq quality. According to the results of the pre-experiment, Hae III was selected to digest the genomic DNA. The enzyme digestion efficiency of control genome *Oryza sativa* ssp. *japonica* was 94.12%, indicating the enzyme digestion reaction was normal. A total of 934.08 MB paired-end reads were obtained, with an average Q30 of 91.97% (89.04–93.44%) and a GC content of 43.67% (42.13–45.56%) (Supplementary Table 2). The mapping rate and the proper mapped rate were 98.20 and 90.96%, respectively (Supplementary Table 3).

**Figure 3:**
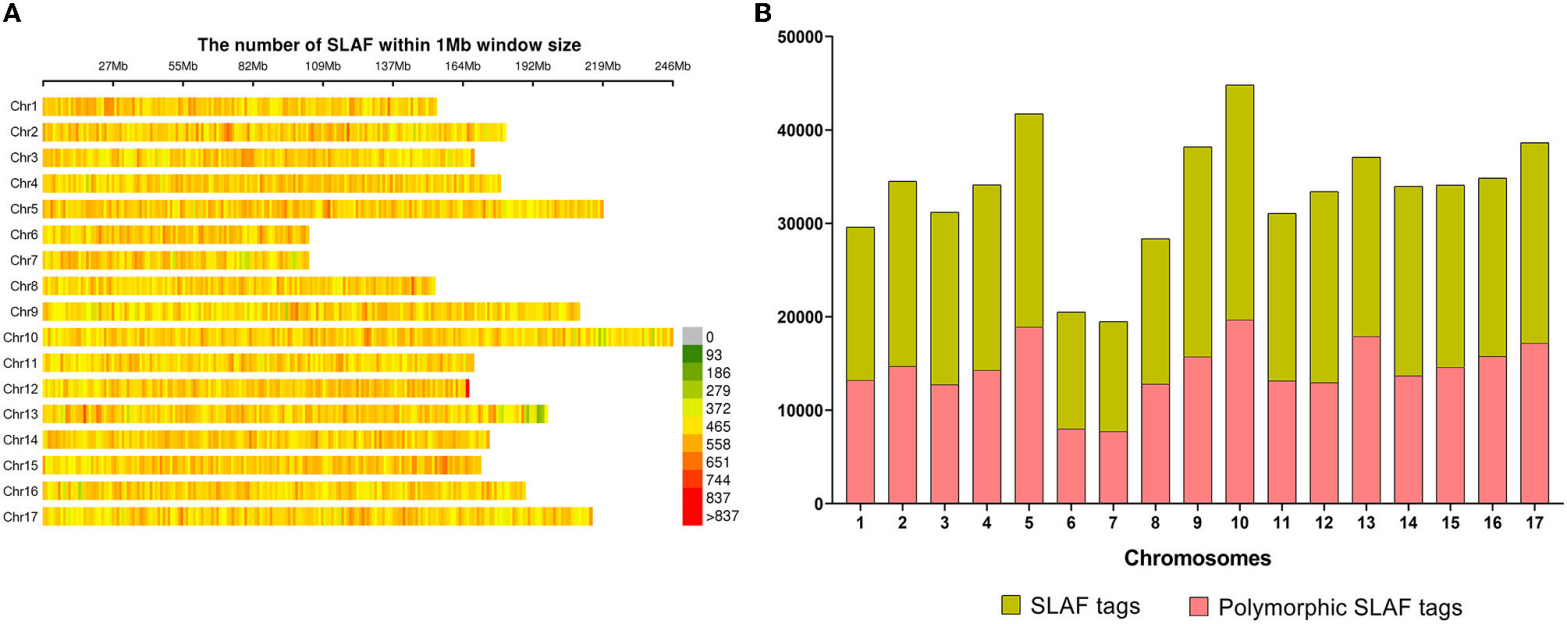
Specific length amplified fragments (SLAF) tags distribution. **(A)** Distribution of all 565,668 SLAF tags on sunflower genome based on 226 accessions. The colors indicate the number of SLAF tags within a 1 Mb window. **(B)** The number of SLAF tags and polymorphic SLAF tags on each chromosome.

**Figure 4:**
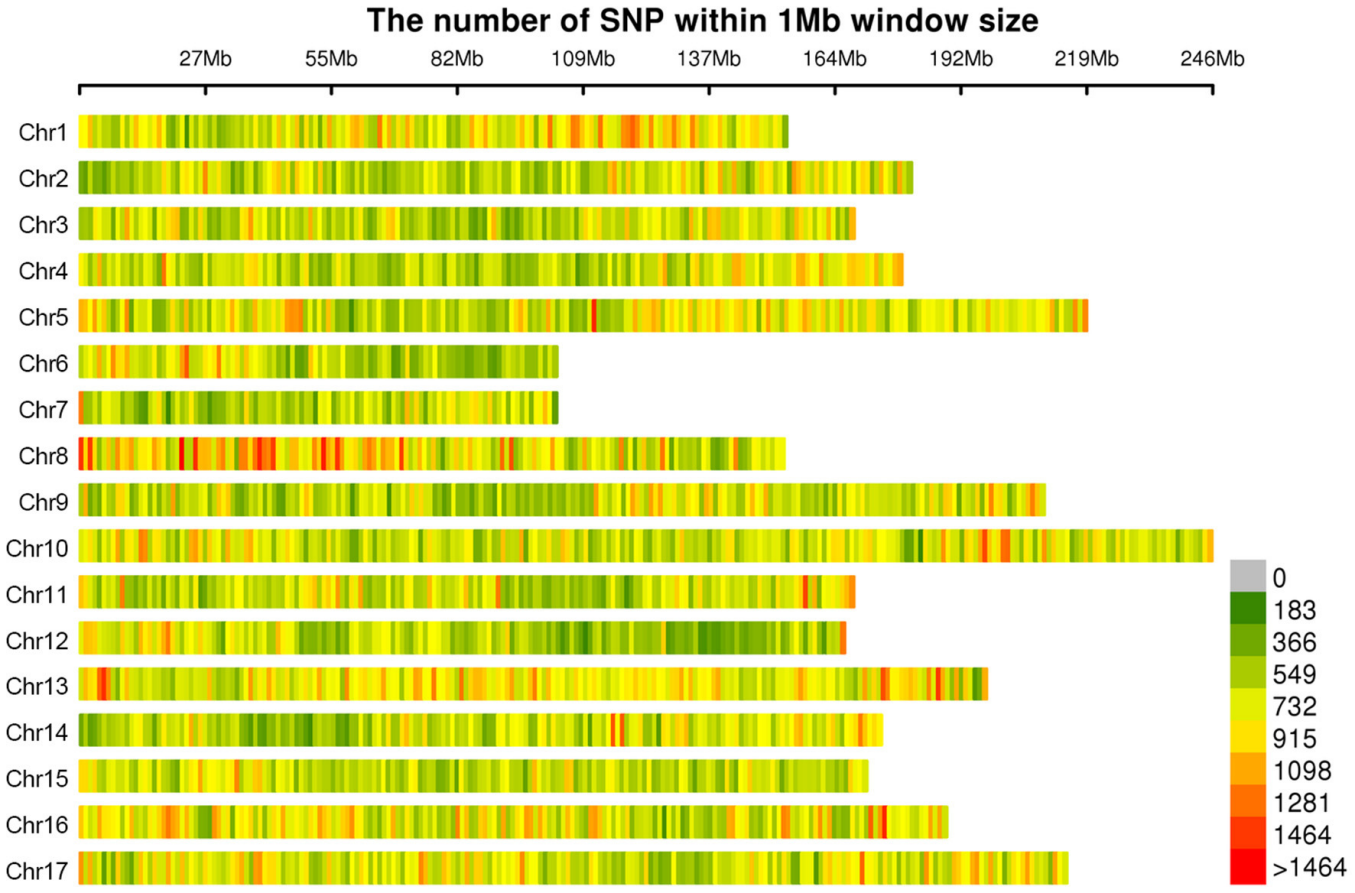
Distribution of all 2,124,143 single nucleotide polymorphisms (SNPs) on sunflower genome. The colors indicate the number of SNPs within a 1 Mb window.

**Figure 5:**
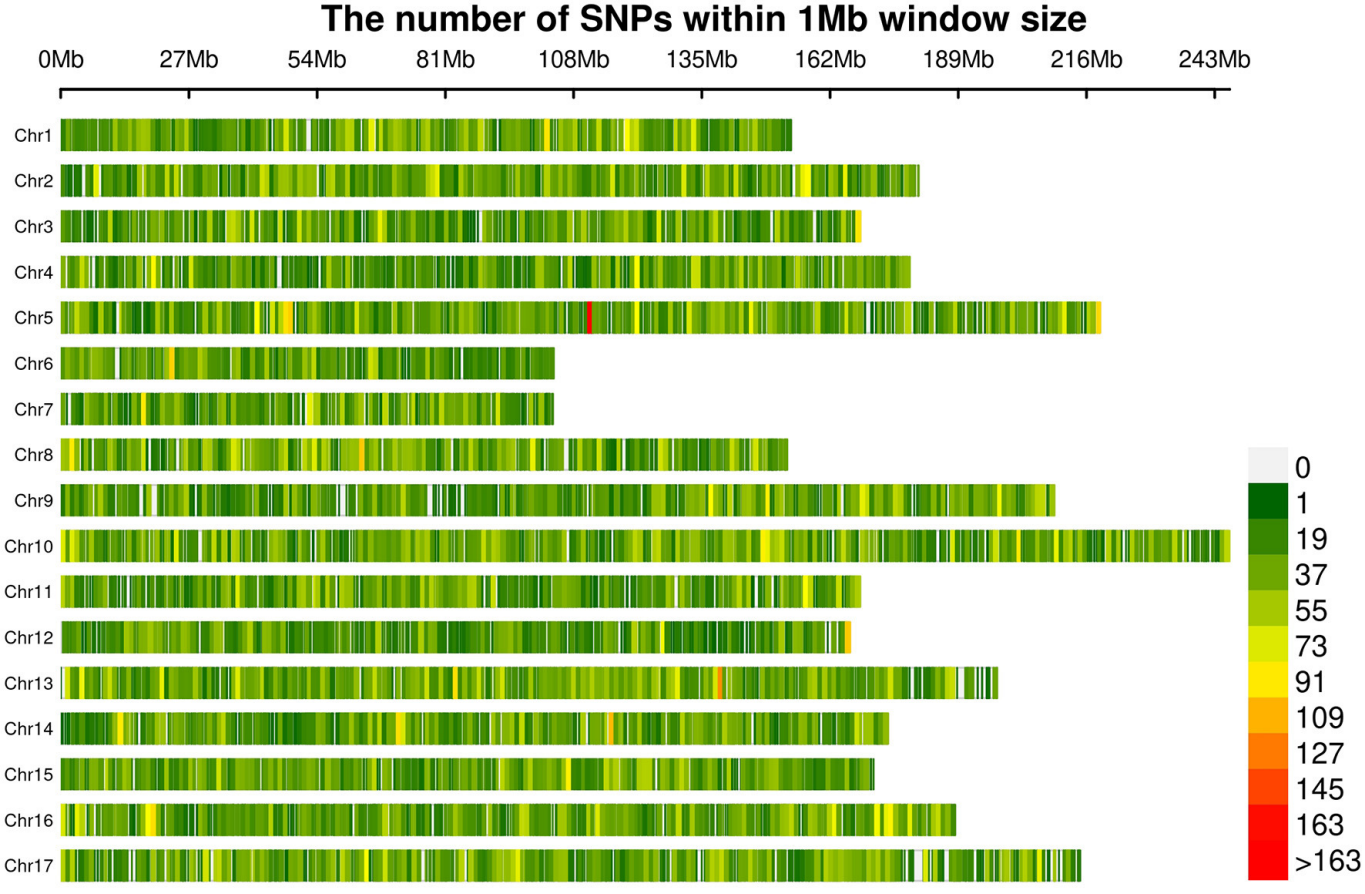
Distribution of filtered SNPs among the 17 chromosomes. The colors indicate the number of SNPs within a 1 Mb window.

**Figure 6:**
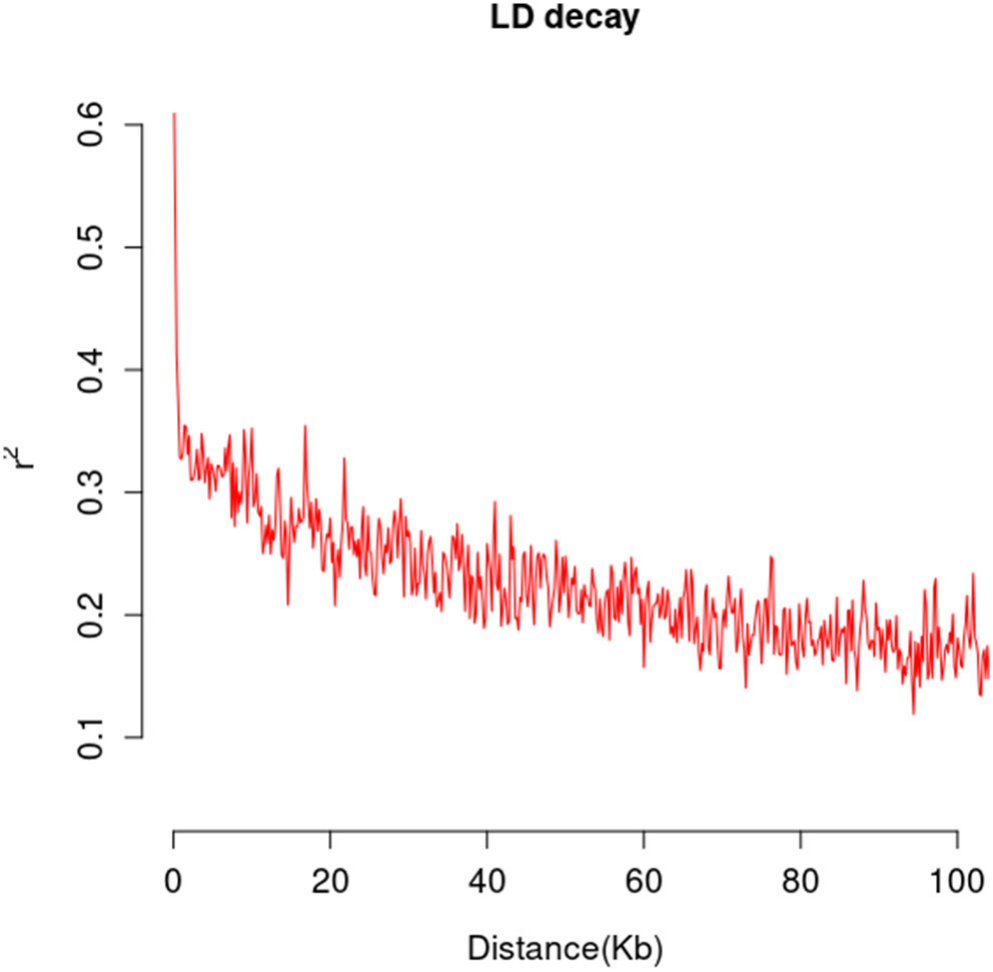
The mean LD decay rate was estimated by the squared allele frequency correlations (*r*^2^) using all pairs of SNPs located within 100 kb of physical distance in genomic regions in a population of 226 sunflower accessions.

A total of 565,668 SLAF tags were obtained, 243,291 of them were polymorphic SLAF tags. These SLAF-tags were evenly distributed on 17 chromosomes (Figure 3; Supplementary Table 4). SLAF tags on chromosome 13 had the highest polymorphic rate (48.25%), while chromosome 12 had the lowest polymorphic rate (38.85%). A total of 2,124,143 population SNP markers were developed *via* GATK software (Supplementary Table 5; Figure 4). After quality control, 94,162 high-quality SNPs were obtained for subsequent analysis (Supplementary Table 6; Figure 5). Chromosome 10 harbored the highest proportion of SNPs (8.68%, 8,173 of 94,162), while the shortest chromosome 6 contained the lowest proportion of SNPs (3.08%, 2,898 of 94,162). There were 31.37 SNP per 1 MB on average across 17 chromosomes. Chromosome 10 had the highest SNPs/Mb ratio (47.68 SNPs per Mb), while chromosome 6 had the lowest SNPs/Mb ratio (19.56 SNPs per Mb) (Supplementary Table 6). LD was estimated as the *r*^2^ value, *r*^2^ ranged from 0.135 on chromosome 6 to 0.218 on chromosome 10, with an average of 0.174, revealing differences in the level of LD among chromosomes (Supplementary Table 7). The average distance of LD decay was about 20 kb (Figure 6).

### Genetic Diversity and Population Structure

**Figure 7:**
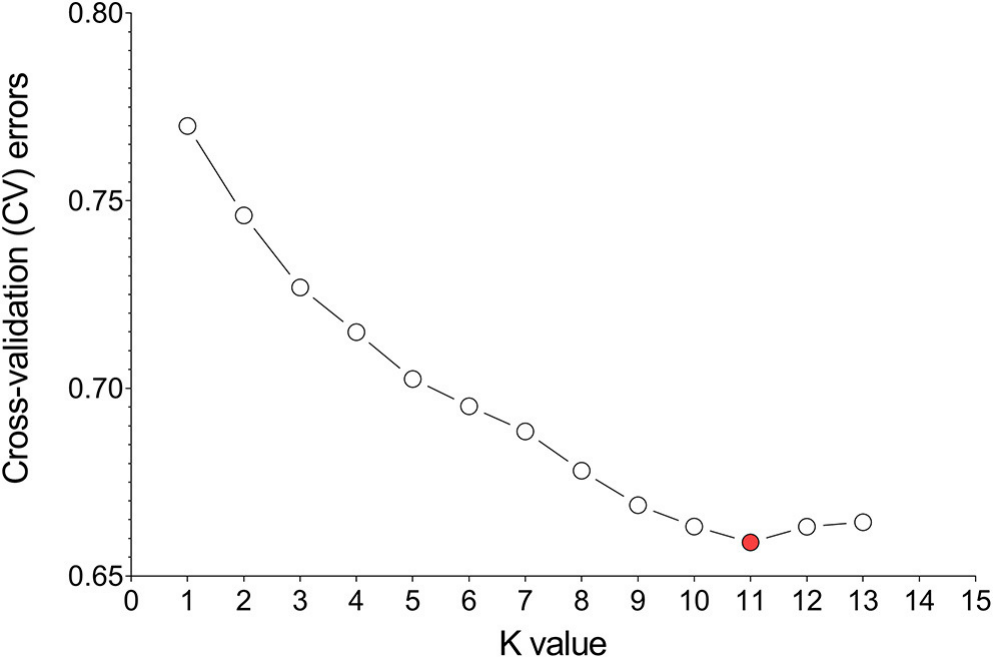
Estimation of cross-validation (CV) errors for K values ranging from 2 to 13. The CV errors declined rapidly from K = 2 ~ 11 and reached the lowest value at K = 11.

**Figure 8:**
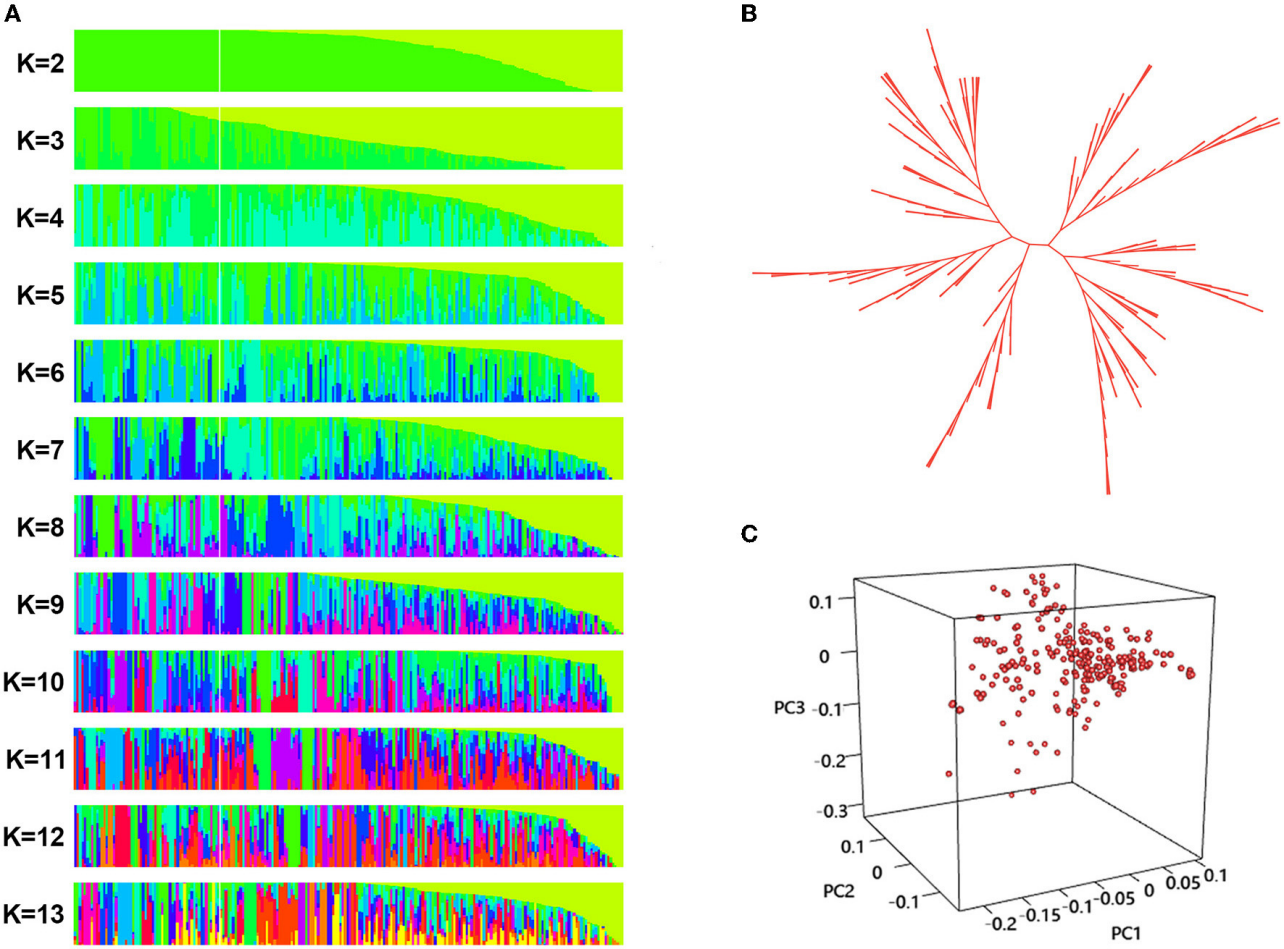
Population structure analysis phylogenetic tree construction, and principal component analysis (PCA) of the 226 sunflower accessions. **(A)** Population structure of sunflower accessions estimated by ADMIXTURE, each row represents a given number of clusters (*K, K* = 2–13), each vertical column represents one individual and each colored segment in each column represents the percentage of the individual in the population. **(B)** The unrooted neighbor-joining three a of 226 sunflower accessions. Each branch indicates a sample, and the length of the branches represents the genetic distance, **(C)** PCA scatter plots shows the distribution of 226 sunflower accessions defined by the eigenvectors of the first three principal components (PC). The three axes represent PC1, PC2, and PC3 respectively. Each dot represents a sample.

Divergence of the 226 accessions during evolution was the major factor leading to high rates of false positive errors in GWAS analysis (Yu and Buckler, 2006). The admixture software was used to analyze the population structure, and the CV for *K* = 1–13 was examined. The results showed that when *K* = 11, the CV dropped to the lowest value (0.659), suggesting the entire population most likely originated from 11 ancestors (Figures 7, 8A). The phylogenetic tree has divided the accessions into 7 main clusters with identical tree topologies (Figure 8B). PCA analysis revealed that all the 11 principal components had eigenvalues of over 1, and the first 8 principal components can explain 85.73% of the total variance. The first three principal components PC1 (with variance explain 15.71%), PC2 (with variance explain 13.55%), and PC3 (with variance explain 11.77%) were displayed in Figure 8C. All these results showed that our experimental materials are highly heterogeneous and is ideal for GWAS analysis.

### Genome-Wide Association Analysis

The GWAS was performed on 8 traits using 3 methods (MLM, FarmCPU, BLINK). A total of 80 SNPs were detected under the significance threshold of *p* < 1.062 × 10^−6^. Among them, 59 were obtained by STI, and 22 were obtained by SSI, and there was only one common SNP between the two indicators (Supplementary Figures 2, 3; Supplementary Table 8). A total of 19, 44, and 33 SNPs were discovered by MLM, FarmCPU, and BLINK methods, respectively. For 8 phenotypic traits, LSA detected the most associated SNPs (27), followed by RWC detected 13, SPAD, RSR, and PH detected 12, 11, and 11, respectively. RL, RV, and RSA were detected 2, 4, and 3 SNPs, respectively. A total of 118 genes were found within the 20 kb of 80 significant SNPs, 85 of them were protein-coding genes (Supplementary Table 9).

### RNA-Sequencing and Expression Analysis

**Figure 9:**
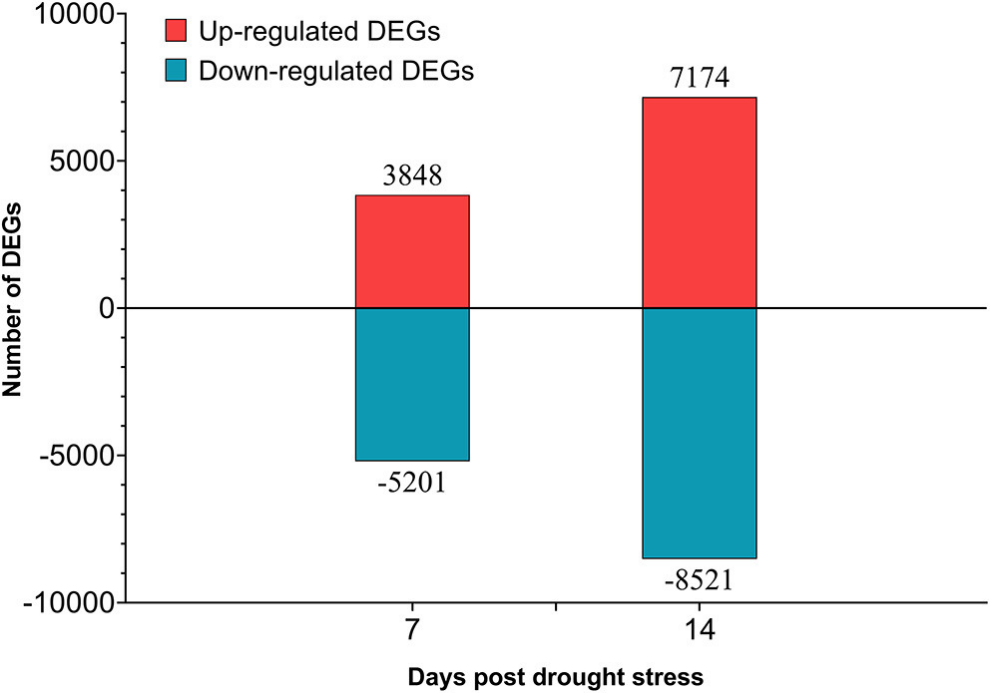
Number of differentially expressed genes (DEGs) in different drought stress time.

A total of 70 Gb clean data were obtained after filtering and quality control. The Q30 of each library ranged from 93.57 to 94.97%, and the GC content ranged from 44.86 to 45.68% (Supplementary Table 10). A total of 18,922 DEGs were obtained (Supplementary Table 11), 6,698 of them were newly discovered. In general, there were more DEGs under 14 days of drought stress compared with the 7 days, and down-regulated DEGs were more than up-regulated DEGs (Figure 9). From day-7 to day-14, the up-regulated DEGs were increasing from 3,848 to 7,174, whereas the down-regulated DEGs were increasing from 5,201 to 8,521, respectively.

### Enrichment Analysis

#### GO Analysis

The up-regulated genes were enriched in 46, 90 GO-terms at 7, 14 days. On day-7, the most significant GO-terms were cellular amino acid catabolic process (GO:0009063), branched-chain amino acid catabolic process (GO:0009083), and seed maturation (GO:0010431). On day-14, the most significant GO-terms were leaf senescence (GO:0010150), aging (GO:0007568), and carboxylic acid catabolic process (GO:0046395). For down-regulated genes, there were 127, and 199 GO-terms enriched at 7, 14 days. On day-7, the most significant GO-terms were cellular polysaccharide metabolic process (GO:0044264), cell wall biogenesis (GO:0042546), and photosynthesis, light reaction (GO:0019684); At day-14, the most significant GO-terms were photosynthesis (GO:0015979), photosynthesis, light reaction (GO:0019684), and plastid organization (GO:0009657) (Supplementary Figure 4).

#### KEGG Analysis

Up-regulated genes were enriched in 13 and 48 significant KEGG pathways at 7 and 14 days. On day-7, the most significant pathways were Valine, leucine and isoleucine degradation, MAPK signaling pathway—plant, and FoxO signaling pathway; On day-14, the most significant pathways were valine, leucine, and isoleucine degradation, MAPK signaling pathway—plant, and longevity regulating pathway. For down-regulated genes, there were 36, 48 significant KEGG pathways enriched at 7, 14 days. On day-7 and day-14, the most significant KEGG pathways were both related to photosynthesis, such as photosynthesis proteins (BR:ko00194), photosynthesis-antenna proteins, and photosynthesis (Supplementary Figure 5).

### RT-qPCR Validation

**Figure 10:**
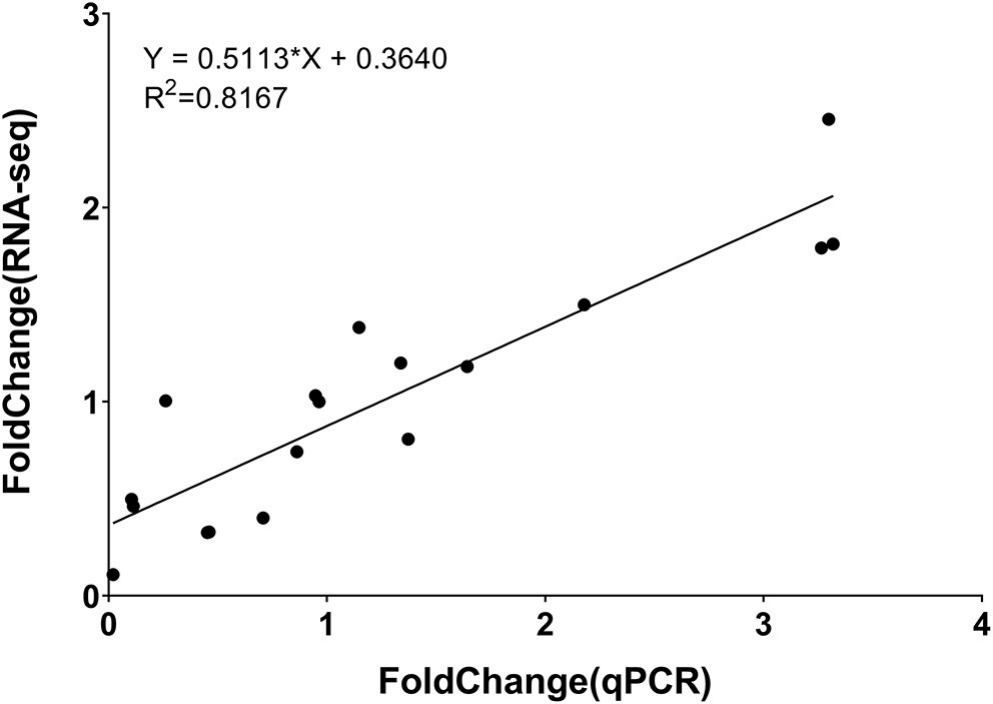
Correlation between results of RT-qPCR and RNA-seq for select DEGs.

To validate the accuracy of RNA-seq, RT-qPCR was performed. Six genes were randomly selected from all DEGs. The primer sequence was shown in Supplementary Table 12. Correlation analysis showed that RNA-seq was closely related to RT-qPCR results. The correlation coefficient (*R*^2^) was 0.8167, endorsing our RNA-seq data were reliable (Figure 10; Supplementary Figure 6).

### Candidate Genes Identification

**Figure 11:**
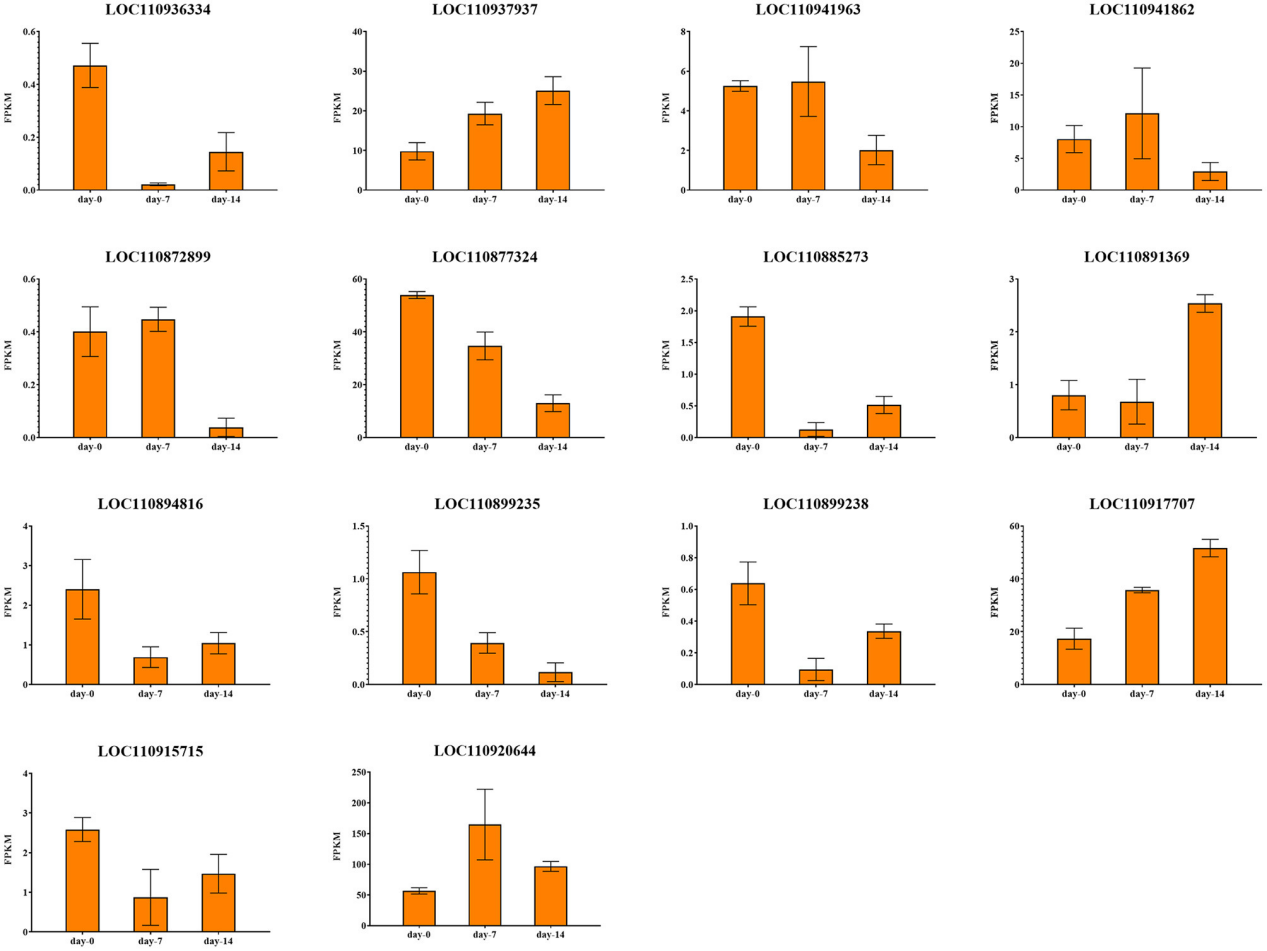
Expression profiles of 14 drought response candidate genes.

By integrating the results of GWAS and RNA-seq analysis, a total of 18 common genes were obtained, 14 of them were protein-coding genes (Table 2; Figure 11). These genes are distributed on chromosomes 4, 5, 8, 9, 10, 11, 12, 13, 16, and 17. Two genes are associated with both LSA and PH. One gene is associated with both LSA and SPAD. Their details are as follows.

**Table 2:** Detail information of 14 genes obtained by combine-analysis of GWAS and RNA-seq.

**Traits**	**Gene name**	**Chromosome**	**Gene_start**	**Gene_end**	**Description**	**iTak**	**Families**
(PH/LSA)-STI	LOC110899235	13	138759411	138769673	Inosine-uridine preferring nucleoside hydrolase		
	LOC110899238	13	138795923	138801286	ABC transporter C family member 3-like		
(LSA/SPAD)-(SSI/STI)	LOC110885273	10	123870286	123873341	Serine threonine-protein kinase	PK	RLK-Pelle_SD-2b
LSA-SSI	LOC110894816	12	55570908	55573165	Equilibrative nucleotide transporter		
	LOC110936334	4	60470023	60472767	Jacalin-like lectin domain		
	LOC110941963	5	200316650	200319863	Microtubule-associated protein RP EB family member		
	LOC110891369	11	160106964	160111888	Receptor-like protein kinase	PK	RLK-Pelle_CrRLK1L-1
	LOC110920644	17	8881157	8883452	PLATZ transcription factor	TF	PLATZ
RSR-SSI	LOC110937937	4	169924932	169927583	Component of the peroxisomal and mitochondrial division machineries. Plays a role in promoting the fission of mitochondria and peroxisomes		
	LOC110915715	16	39633096	39638580	Protein of unknown function (DUF1666)		
RV-STI	LOC110877324	9	29925998	29927713	Belongs to the UDP-glycosyltransferase family		
	LOC110917707	16	74795170	74799439	Domain present in VPS-27, Hrs and STAM		
RSA-STI	LOC110872899	8	68366663	68376805	Inactive leucine-rich repeat receptor-like serine threonine-protein kinase	PK	RLK-Pelle_LRR-III
RWC-SSI	LOC110941862	5	195719872	195730082	Topless-related protein		

*The content in brackets indicates simultaneous, for example, (PH/LSA)-STI, indicating that this gene is recognized by both PH-STI and LSA-STI*.

#### Candidate Genes Associated With Plant Height

There were 2 candidate genes that were screened using combined analysis. Both of them were located on chromosome 13. The LOC110899235 gene encoding “inosine-uridine preferred nuclear hydrate” is homologous to the AT5G18860.2 gene in *Arabidopsis thaliana*. Another LOC110899238 gene encoding “ABC transporter c family member 3-like” is homologous to the AT3G13080.1 gene in *Arabidopsis thaliana*. Both two genes were down-regulated with the extension of drought stress time in K58.

#### Candidate Genes Associated With Leaf Surface Area

There were 8 common candidate genes associated with LSA, 2 of which were also associated with PH. The function of the gene LOC10936334 located on chromosome 4 was annotated as “Jacalin-like lectin domain”, which is homologous to the AT1G73040.1 gene in *Arabidopsis thaliana*, and its expression level continues to decrease under drought stress in K58. Gene LOC110941963 located on chromosome 5 was annotated as “microtubule-associated protein RP EB family member”, which was homologous to the AT3G47690.1 gene in *Arabidopsis thaliana*. This gene was down-regulated after 14 days of drought stress in K58. At 19.52 kb upstream of an SNP (S10_123892851) on chromosome 10, a gene (LOC110885273) encoding “Serine threonine-protein kinase” was identified. It is worth noting that the gene was also associated with SPAD. This gene belongs to the protein kinase family of RLK-Pelle_SD-2b, and is homologous to the *Arabidopsis* AT4G32300.1 gene. RNA-seq showed it was down-regulated with the extension of drought stress in K58. Gene LOC110894816 encoding “Equilibrative nucleotide transporter” were down-regulated at 7, 14 days in K58, which is homologous to AT1G70330.1 in *Arabidopsis thaliana*. Gene LOC110920644 belongs to the PLATZ transcription factor family. It was up-regulated at 7 days and down-regulated at 14 days of drought stress in K58. Gene LOC110891369 encoding “receptor-like protein kinase” was sharply up-regulated at 14 days. This protein kinase belongs to the RLK-Pelle_SD-2b RLK-Pelle_CrRLK1L-1 protein kinase family.

#### Candidate Genes Associated With Root-Shoot Ratio

There were 2 candidate genes obtained by combined analysis. One gene LOC110937937 encoding “Component of the peroxisomal and mitochondrial division machineries” was up-regulated at 14 days post drought stress, another gene LOC110915715 encoding “Protein of unknown function (DUF1666)” were continuously down-regulated with the drought stress.

#### Candidate Genes Associated With Three Root Related Traits

Notably, there are relatively fewer SNPs related to three root traits (RL, RV, and RSA). No genes were found within the 20 kb region of RL associated SNPs. The combined analysis identified 2 genes associated with RV and 1 gene associated with RSA. For RV, gene LOC110877324 on chromosome 9 was annotated as “Belongs to the UDP-glycosyl transferase family”, which was down-regulated in K58 after 14 days of drought stress. Another gene (LOC110917707) located on chromosome 16 was annotated as “domain presence in VPS-27, Hrs and Stam”, which was up-regulated in K58 after 14 days of drought stress. These two genes are homologous to the AT2G18570.1 gene and AT2G38410.1 gene in *Arabidopsis thaliana*, respectively.

For RSA, gene LOC110872899 was located on chromosome 8, and annotated as “Inactive leucine-rich repeat receptor-like serine threonine-protein kinase”. This gene is homologous to the *Arabidopsis* AT1G10850.1 gene. It was slightly up-regulated in K58 at 7 days and then sharply down-regulated at 14 days of drought stress.

#### Candidate Genes Associated With Relative Water Content

LOC110941862 is the unique gene screened by the combined analysis. This gene encodes the “Topless-related protein”, which is homologous to the AT1G15750.3 gene in *Arabidopsis thaliana*. RNA-seq results showed that this gene was continuously down-regulated in K58 under drought stress.

## Discussion

Global climate change threatens crop production worldwide. Plants adopt diverse strategies to combat drought stress such as reducing the stomatal conductance, decreased photosynthetic rate, accumulation of different osmoprotectants, activation of stress-responsive genes and transcription factors, etc. (Farooq et al., 2009; Kaur and Asthir, 2017). Drought resistance is a complex quantitative trait. One difficulty in drought-tolerant genetic breeding is the unequivocal evaluation of plant response to soil-water deficits (Pereyra-Irujo et al., 2007). Based on the previous research, we evaluated 8 phenotypic traits among 226 accessions under WW and DS conditions. Compared to the WW condition, the average PH, LSA, RWC, and SPAD value were decreased, while RSR and three root related traits (RL, RV, RSA) were increased under the DS condition.

It has long been known that drought stress at the vegetative stage impedes phenotypic traits like PH, LSA, whereas an increase in RL at the expense of above-ground dry matter occurs resulting in higher RSR (Petcu et al., 2001; Hussain et al., 2010; Javaid et al., 2015). In our results, the change trends of mean PH, RL, RSR, and LSA were consistent with previous studies. However, the mean RV increased under drought, which was not consistent with a previous study. Geetha et al. (2012) found that the RV decreased by 40.2% under drought stress among 29 sunflower varieties, while we found 83% of accessions have an increase in RV. This may be due to differences in the genotypes of the study materials. Different genotypes of plants have different adaptability to drought stress (Petcu et al., 2001). Even in the most consistent trend of variation in PH (92% decreased under drought stress), there were still 16 accessions increased under drought stress. These specific materials may include important drought-tolerance genes and will be good sources for our drought tolerance molecular breeding. In some previous studies, the relationship between SPAD and chlorophyll content per unit leaf area is fitted as linear regression. SPAD value is often used to represent chlorophyll content (Costa et al., 2001; Martínez and Guiamet, 2004). Our results show that under WW growth conditions, SPAD value is positively correlated with LSA. It demonstrates that a larger LSA has more chlorophyll, which increases the photosynthetic rate (Espina et al., 2018). The correlation coefficients of LSA and SPAD in WW vs. DS conditions were higher than 0.6, indicating that drought affects these two traits more by environment than by genotype. The correlation coefficients of RSA, RL and RL were very low, indicating that they were more influenced by genotype.

Studies have shown that the genetic relatedness of the mapping population can increase the false positive risk of GWAS results (Ali et al., 2020). A population with enough genotype and trait diversity is considered to be the expected GWAS population (Flint-Garcia et al., 2005). In this study, the population panel consisting of 226 accessions were collected from different ecological regions. Three population structure analysis methods (admixture, phylogenetic, and PCA) were conducted. Results showed that 226 sunflower materials had large genetic differences and were an ideal GWAS population. Linkage disequilibrium (LD) is the basis of GWAS (Ali et al., 2020). When LD declines rapidly with distance, LD mapping is potentially very precise (Gaut and Long, 2003). Since our materials have high genetic variability, the LD-decay distance is about 20 kb. Overall patterns of LD decay show chromosome specificity. Chr10 showed the highest LD value, followed by Chr7, Chr5, Chr13, and Chr17. This result is consistent with a previous study conducted by Filippi et al. (2020). They have reported different patterns of LD across chromosomes, with Chr10, Chr17, Chr5, and Chr2 showing the highest LD. The extended LD in Chr10 and Chr5 were also reported by other researchers (Cadic et al., 2013; Mandel et al., 2013). Owens et al. showed that the extended LD on Chr10 could be the result of the wild introgression in the fertility restoring male lines (Owens et al., 2019).

GWAS methods have evolved over years. Several new methods are being developed to improve the statistical power and reduce the computational time. FarmCPU uses a set of markers associated with a casual gene as a co-factor instead of kinship to avoid overfitting and eliminate confounding between kinship and testing markers iteratively (Liu et al., 2016). More recently, along with improvements in statistical power and reduction in computing time compared to FarmCPU, the new method called BLINK is set to eliminate FarmCPU requirement that quantitative trait nucleotides (QTNs) are evenly distributed in the genome (Huang et al., 2019). In the present study, we used 3 methods simultaneously to conduct GWAS. The FarmCPU method detected 44 SNPs, the BLINK method detected 33 SNPs, and the MLM method detected the lowest of 19 SNPs, respectively. There were 12 SNPs found simultaneously by FarmCPU and BLINK method, and only 3 common SNPs were found by 3 methods. Most SNPs were only found in one method. Therefore, it may be prudent to use multiple methods to conduct a GWAS survey (Nida et al., 2021).

STI and SSI are two commonly used evaluation indexes in the study of plant abiotic stress. According to the research of Mehdi GHAFFARI, STI is more efficient for identifying drought-resistant lines, and SSI is more efficient for identifying drought-sensitive lines (Ghaffari et al., 2012). Applying both indicators simultaneously could provide a complete and accurate assessment of drought tolerance. Strangely, the calculation methods of STI in different articles are inconsistent (Sukumaran et al., 2018; Khanzada et al., 2020; Chaurasia et al., 2021). In the present study, we carefully chose a scientific STI calculation method for GWAS analysis. A total of 80 significant SNP markers associated with 8 phenotypic traits were detected, 22 of them were detected using SSI, and 59 of them were detected using STI, only one common SNP was detected by both of the two indexes.

To further understand the biological processes, pathways, and gene expression patterns in sunflowers under drought stress, we conducted an RNA-seq analysis. Based on the phenotypic traits, a drought-tolerant plant was selected from the GWAS population. We sampled the leaves at 0, 7, and 14 days after drought stress. A total of 18,922 differentially expressed genes were obtained.

There was a noticeable consistency between the results of GO and KEGG analysis. For example, up-regulated genes were enriched in GO-terms such as cellular amino acid catabolic process (GO:0009063), branched-chain amino acid catabolic process (GO:0009083), while KEGG analysis showed “Valine, leucine and isoleucine degradation” was the most significant pathway. Down-regulated genes were enriched in photosynthesis (GO:0015979), photosynthesis, light reaction (GO:0019684) according to GO analysis, while KEGG analysis showed down-regulated genes enriched in pathways such as Photosynthesis proteins (BR:ko00194), Photosynthesis—antenna proteins, Photosynthesis. The branched-chain amino acids (BCAAs), including isoleucine, leucine, and valine, are essential for plants (Binder et al., 2007). Pires et al. (2016) results highlight that catabolism of BCAA appears to play an important role in the mechanism of tolerance to short-term drought, most likely by delaying the onset of stress. Our results also proved that the degradation of BCAA may be an important mechanism of sunflower drought resistance. Abiotic stress damage the thylakoid membrane, disturb its functions, and ultimately decrease photosynthesis. Down-regulated expression of photosynthesis-related genes under drought stress has been reported in several plants, such as *Arabidopsis* (Bechtold et al., 2016; Bouzid et al., 2019), wheat (Derakhshani et al., 2020), and grapevines (Franck et al., 2020). In a previous study, Escalante et al. found a down-regulation of photosynthesis-related genes in the aerial part of sunflowers (Moschen et al., 2017). However, another study revealed that the expression levels of photosynthesis-related genes were increased under drought stress in sunflowers (Escalante et al., 2020). This difference may be caused by differences in drought intensity and genotype, and our results were identical with the former.

With the development of high-throughput technologies, omics research is also undergoing a shift from a single-omics to a large-scale multi-omics approach (Liu et al., 2020). Through the multi-omics approach, researchers can obtain a deeper understanding of the fundamental biological processes, a more accurate prediction of the response variable, and gain further insight into mechanistic aspects of the system (Cavill et al., 2015). By integrating the transcriptome and metabolome, Sebastián Moschen et al. (2017) gained a deeper insight into the sunflower drought-response mechanism. The integration of genomic and transcriptomic analysis has also been reported in many recent studies. This approach can be used as an effective way to identify candidate genes. For example, eight salt stress-related candidate genes were identified by a combination of GWAS analysis and transcriptome analysis in Alfalfa (*Medicago sativa* L.) (He et al., 2021). Seven candidate genes for seminal root length in maize (*Zea mays* L.) were identified by integrating the results of the GWAS, the common DEGs, and the co-expression network analysis (Guo et al., 2020). Using a combined analysis, we identified 18 common genes.

The total genes in the sunflower reference genome were 81,496, and we found 18,922 DEGs *via* RNA-seq. According to this proportion, we should find at least 29 DEGs among the 118 genes of GWAS. However, the number of common genes that we have found was relatively small (18). This is because among the 18,922 DEGs, only 12,124 of them exist in the reference genome and the rest are novel genes. A subsequent chi-square test using this number found no significant difference between the two proportions (*P* = 0.908). Nonetheless, the proportion of overlapped genes was still lower than we expected. The reason we speculate is that GWAS candidate genes are mainly regulatory genes that act in all accessions. A slight regulation of expression level under drought stress, which did not reach the threshold of significant difference, can affect the physiological processes in plants, whereas the DEGs of RNA-seq are mainly a series of drought-responsive functional genes that are regulated in K58 under drought stress. The difference in the class and function of the genes from these two gene sets results in a low percentage of overlapping genes. Of course, this needs further confirmation.

Among these 18 genes, 14 are protein-coding genes, of which 3 are encoding PK and 1 encodes TF. These genes may play an important role in drought response in sunflowers. The LOC110885273 gene encodes G-type lectin S-receptor-like serine/threonine-protein kinase (LecRLKs). The protein kinase is involved in plant responses to biotic and abiotic stresses (Bonaventure, 2011; Singh et al., 2012; Zhao et al., 2016). Overexpression of G-type LecRLKs enhances the drought tolerance of *Arabidopsis thaliana* (Sun et al., 2013a), which may be achieved by controlling stomata size through interaction with abscisic acid (ABA) (Arnaud et al., 2012). Pan et al. (2020) identified a LecRLKs gene OsESG1 in rice and found it could be induced by treating with PEG, NaCl, and ABA. However, we found the LOC110885273 gene was down-regulated under drought stress, which may lead to the decrease of SPAD value under drought stress.

The receptor like kinase (RLKs) family has been defined as the most abundant gene family in *Arabidopsis*. Leucine rich repeat-RLKs (LRRRLKs) are the largest group of receptor-kinases in Arabidopsis, which is widely involved in responses to various biotic and abiotic stresses (Diévart and Clark, 2003; Lehti-Shiu et al., 2009). Osakabe et al. (2005) found that an LRRRLKs gene (RPK1) is involved in the early steps in the ABA signaling pathway through a gene knock-out experiment. The overexpression of receptor-like kinase rich in the Leucine Repetition gene improves the *Arabidopsis thaliana* drought resistance (Xing et al., 2011). Receptor-like cytoplasmic kinase GUDK and OsSIK1 were shown to enhance drought tolerance in rice (Ouyang et al., 2010; Harb et al., 2020). In the present study, a down-regulated LRRRLKs gene LOC110872899 was identified, which is located at chromosome 8, and associated with RSA, maybe the mechanism of this gene in sunflower drought tolerance response is different. Another receptor-like protein kinase gene LOC110891369 was up-regulated at 14-days of drought stress in K58, which belongs to the family of RLK-Pelle_CrRLK1L-1, and is associated with LSA.

PLATZ transcription factors play important roles in plant growth, development, and biotic and abiotic stress responses. Liu et al. (2021) reveal that PLATZ4 interacts with AITR6 to increase ABA sensitivity and drought tolerance in *Arabidopsis* by regulating the expression of different genes. Zenda et al. (2019) identified a PLATZ gene (Zm00001d051511) in maize. It was up-regulated in tolerant line YE8112, whilst down-regulated in drought-sensitive line after drought stress. This result indicated the TF genes could be the key contributors to drought stress tolerance in the drought-tolerant maize inbred line. This different expression pattern was also proved in Ray's research on rice (Ray et al., 2011), PLATZ (LOC_Os10g42410) gene was down-regulated in panicle, while up-regulated in vegetative tissues under drought stress. Even in the same tissue at the same time, it was found that the expression levels of two PLAZT genes were up-regulated and down-regulated, respectively, which indicated the complexity of drought stress regulation. In this study, a PLAZT gene LOC110920644, which is related with LSA, was up-regulated at the early stage in K58 under drought stress.

ABA is an important hormone for plant drought response (Zotova et al., 2018). The cell ABA level increases under drought stress, leading to stomatal closure and active several stress-responsive genes (Cutler et al., 2010). Drought stress increased ABA levels in sunflowers have been reported (Robertson et al., 1985). In this study, the functions of the four TF/PK genes are all related to ABA, indicating the important role of the ABA-dependent process in the drought response of sunflowers.

## Conclusion

Sunflower is one of the most important oil crops in the world, which is often grown as a rainfed crop. Water limitation at the seedling stage can severely reduce stand establishment and negatively impact yields. However, the molecular mechanism underlying drought resistance is still not fully understood. In this study, we used SLAF-seq to perform GWAS for 8 important phenotypic traits in 226 sunflower inbred lines. Using three methods (i.e., MLM, FarmCPU, and BLINK) for sunflower grown in two conditions (i.e., well-water and drought stress), we identified a total of 80 SNP displaying a significant association (*p* < 1.062 × 10^−6^). Candidate genes were searched in the 20 kb up/down-stream of each SNP. There were 85 protein-coding candidate genes possibly related to the 8 important phenotypic traits. Next, we conducted an RNA-seq based on a drought-tolerance inbred line (K58). A total of 18,922 DEGs were identified on 7 and 14 days after drought treatment. Up-regulated genes were mainly enriched in BCAA catabolic process, while down-regulated genes were mainly enriched in the photosynthesis process. Using a combined analysis, we found 14 common genes between GWAS and RNA-seq, three of them were PK genes, and one of them was TF gene. LOC110885273 was associated with LSA and SPAD, belongs to the RLK-Pelle_SD-2b protein kinase family. LOC110872899 belongs to the RLK-Pelle_LRR-III protein kinase family and is associated with RSA. LOC110891369 belongs to the RLK-Pelle_CrRLK1L-1 protein kinase family and is associated with LSA. The PLAZT gene LOC110920644 is related to LSA, and belongs to PLAZT TF family. Through functional analysis, there are 4 genes involving the ABA-dependent drought response pathway of plants.

The integrative analysis of omics data is a promising approach to identify candidate genes for complex traits. This study is the first attempt to combine GWAS and RNA-seq to explore the genetic mechanism of sunflower drought tolerance to our knowledge. We will further validate the functions of these genes, possibly by overexpression or by CRISPER/Cas genome editing. Our research reveals the phenotypic and molecular mechanisms of drought response in sunflowers. The results will be useful for the genetic enhancement of drought-resistant sunflowers.

## Data Availability Statement

The original contributions presented in the study are publicly available. This data can be found here: https://www.ncbi.nlm.nih.gov/search/all/?term=PRJNA797473.

## Author Contributions

JH and LY: conceptualization. LY, YM, and YW: methodology. YW: software, writing—original draft preparation, and visualization. JH: validation, project administration, and funding acquisition. YW, ZZ, and HS: formal analysis. YM, ZZ, HH, and ZH: investigation. HY: resources. YM and ZZ: data curation. LY, YZ, and HY: writing—review and editing. LY: supervision. All authors have read and agreed to the published version of the manuscript.

## Funding

This work was financially supported by grants from the National Natural Science Foundation of China (Nos. 32160450 and 31760396).

## Conflict of Interest

The authors declare that the research was conducted in the absence of any commercial or financial relationships that could be construed as a potential conflict of interest.

## Publisher's Note

All claims expressed in this article are solely those of the authors and do not necessarily represent those of their affiliated organizations, or those of the publisher, the editors and the reviewers. Any product that may be evaluated in this article, or claim that may be made by its manufacturer, is not guaranteed or endorsed by the publisher.
