# Supplementary figures and images for: Combined GWAS and Transcriptome Analyses Provide New Insights Into the Response Mechanisms of Sunflower Against Drought Stress
Source: Front Plant Sci. 2022 May 3;13:847435. doi: 10.3389/fpls.2022.847435 (PMC9111542; doi:10.3389/fpls.2022.847435)

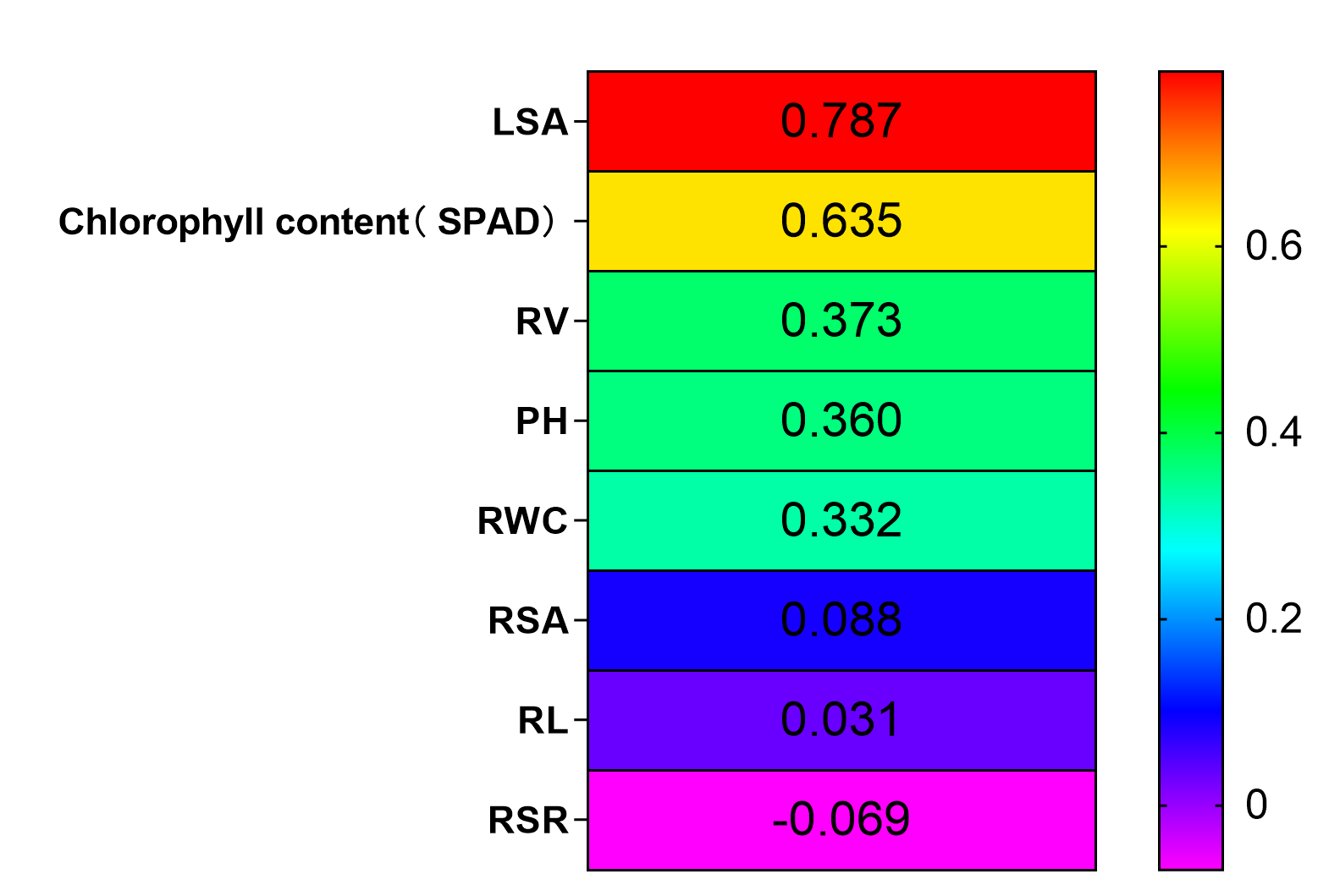

Supplement: Supplementary file 1 [file Image_1.TIF]

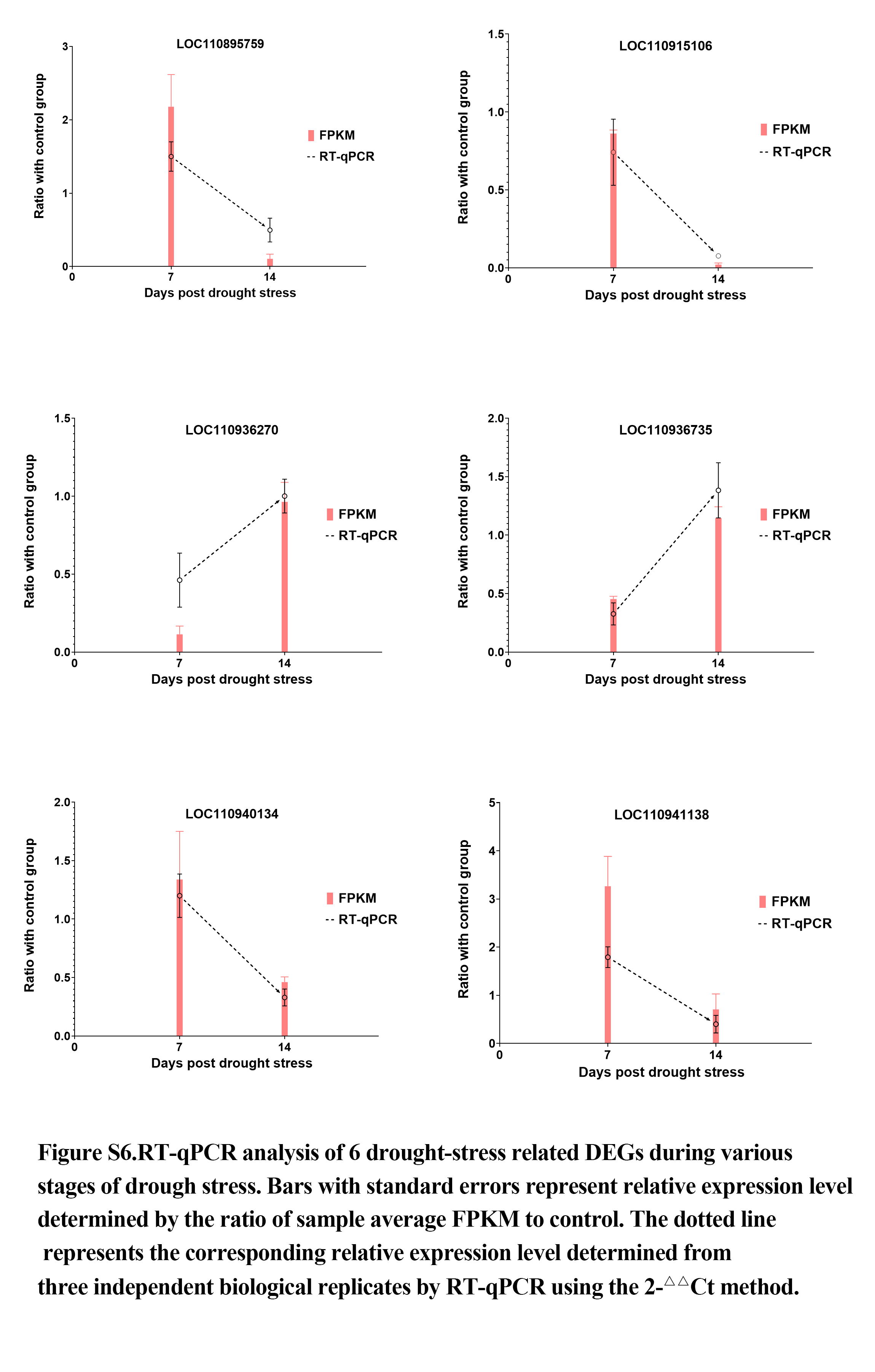

Supplement: Supplementary file 2 [file Image_2.TIF]

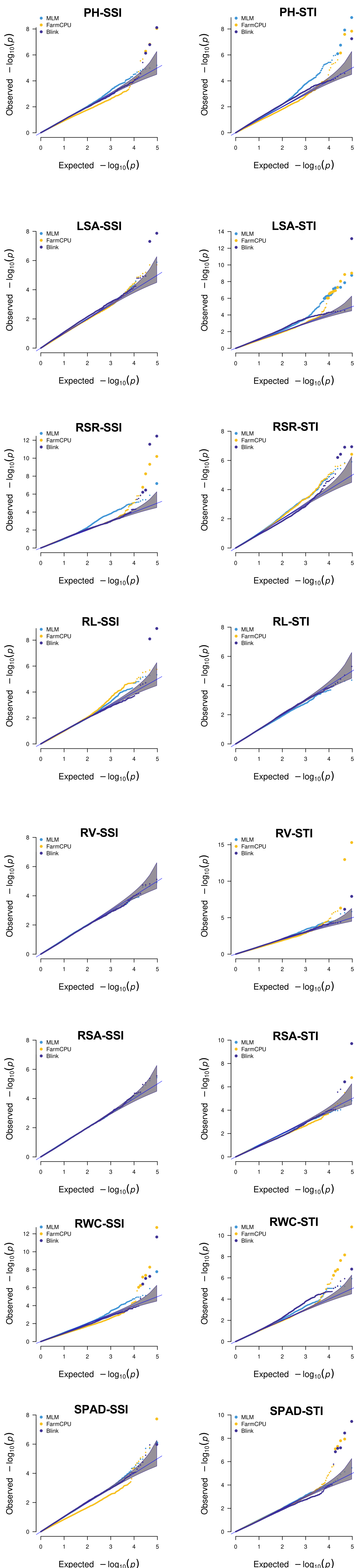

Figure S3. Quantile-quantile (Q-Q) plots from GWAS for 8 drought related traits.

Supplement: Supplementary file 3 [file Data_Sheet_1.PDF]
