# Supplementary material for: Combined GWAS and Transcriptome Analyses Provide New Insights Into the Response Mechanisms of Sunflower Against Drought Stress
Source: Front Plant Sci. 2022 May 3;13:847435. doi: 10.3389/fpls.2022.847435 (PMC9111542; doi:10.3389/fpls.2022.847435)
Supplement: Supplementary file 4 [file Data_Sheet_2.PDF]

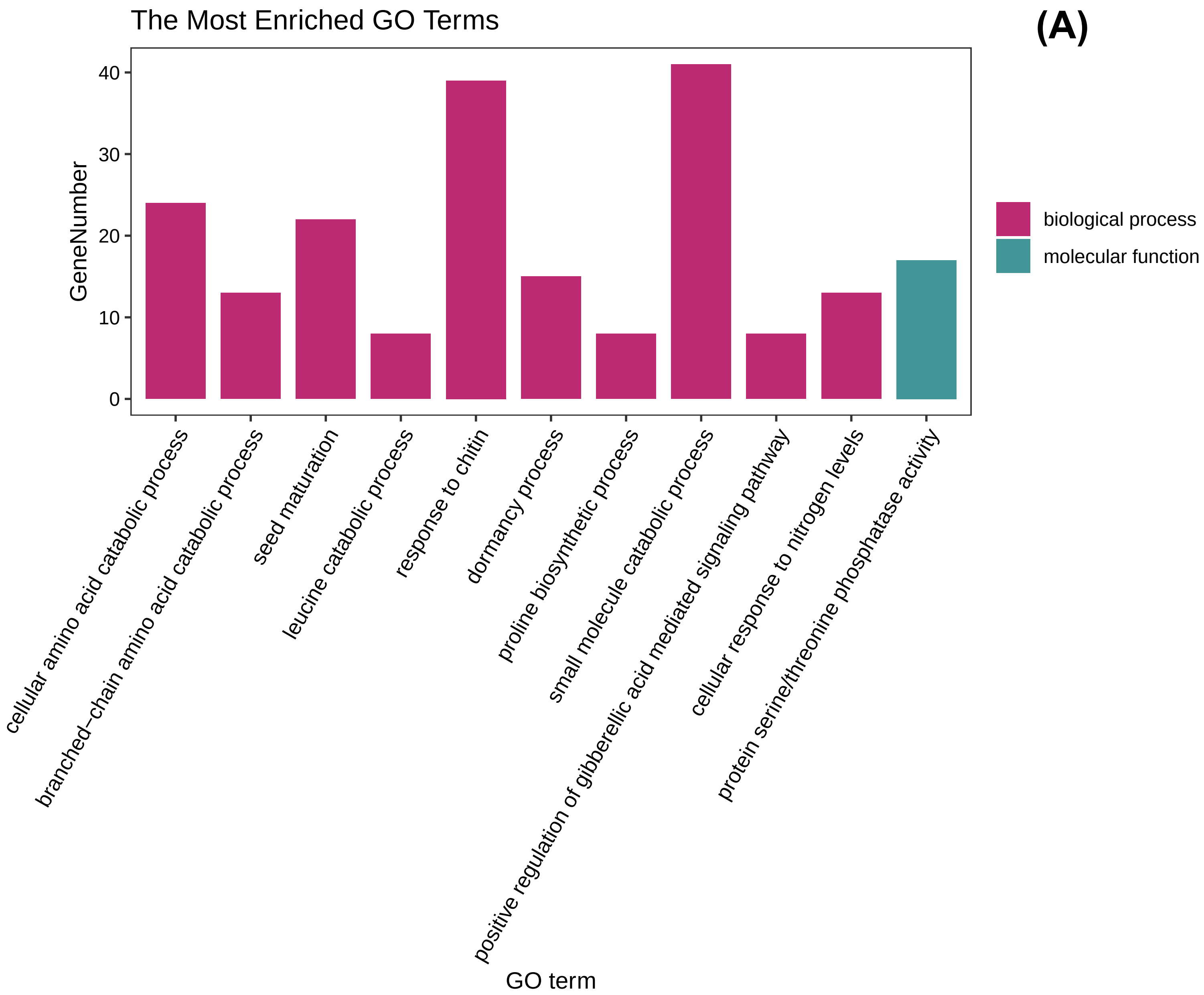
The Most Enriched GO Terms
(A)
GeneNumber
0
10
20
30
40
biological process
molecular function
cellular amino acid catabolic process
branched–chain amino acid catabolic process
seed maturation
leucine catabolic process
response to chitin
dormancy process
proline biosynthetic process
small molecule catabolic process
positive regulation of gibberellic acid mediated signaling pathway
cellular response to nitrogen levels
protein serine/threonine phosphatase activity
GO term

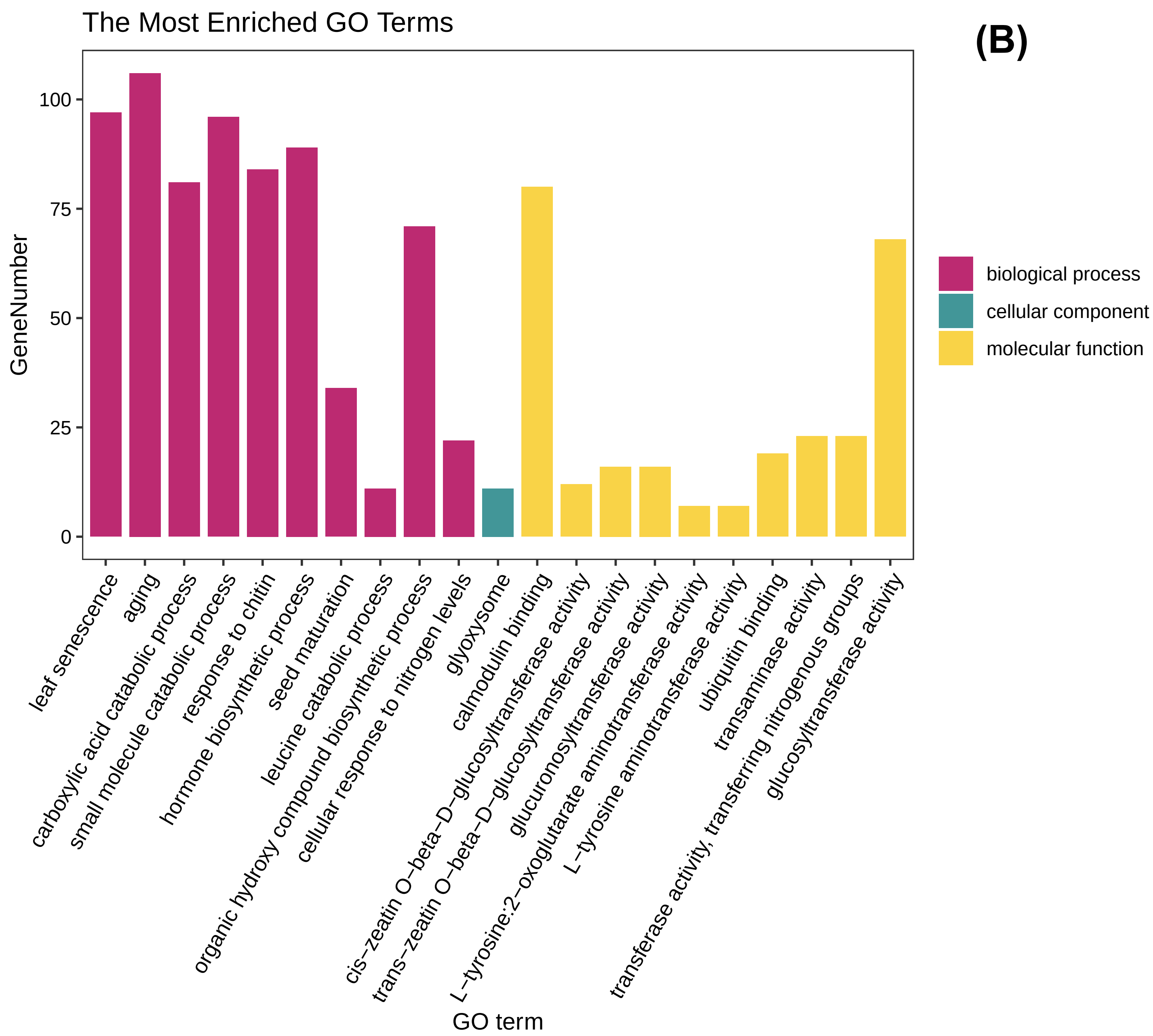

The Most Enriched GO Terms
(B)
GeneNumber
0
25
50
75
100
biological process
cellular component
molecular function
leaf senescence
aging
carboxylic acid catabolic process
small molecule catabolic process
response to chitin
hormone biosynthetic process
seed maturation
leucine catabolic process
organic hydroxy compound biosynthetic process
cellular response to nitrogen levels
glyoxysome
calmodulin binding
cis−zeatin O−beta−D−glucosyltransferase activity
trans−zeatin O−beta−D−glucosyltransferase activity
glucuronosyltransferase activity
L−tyrosine:2−oxoglutarate aminotransferase activity
L−tyrosine aminotransferase activity
ubiquitin binding
transaminase activity
transferase activity, transferring nitrogenous groups
glucosyltransferase activity
GO term

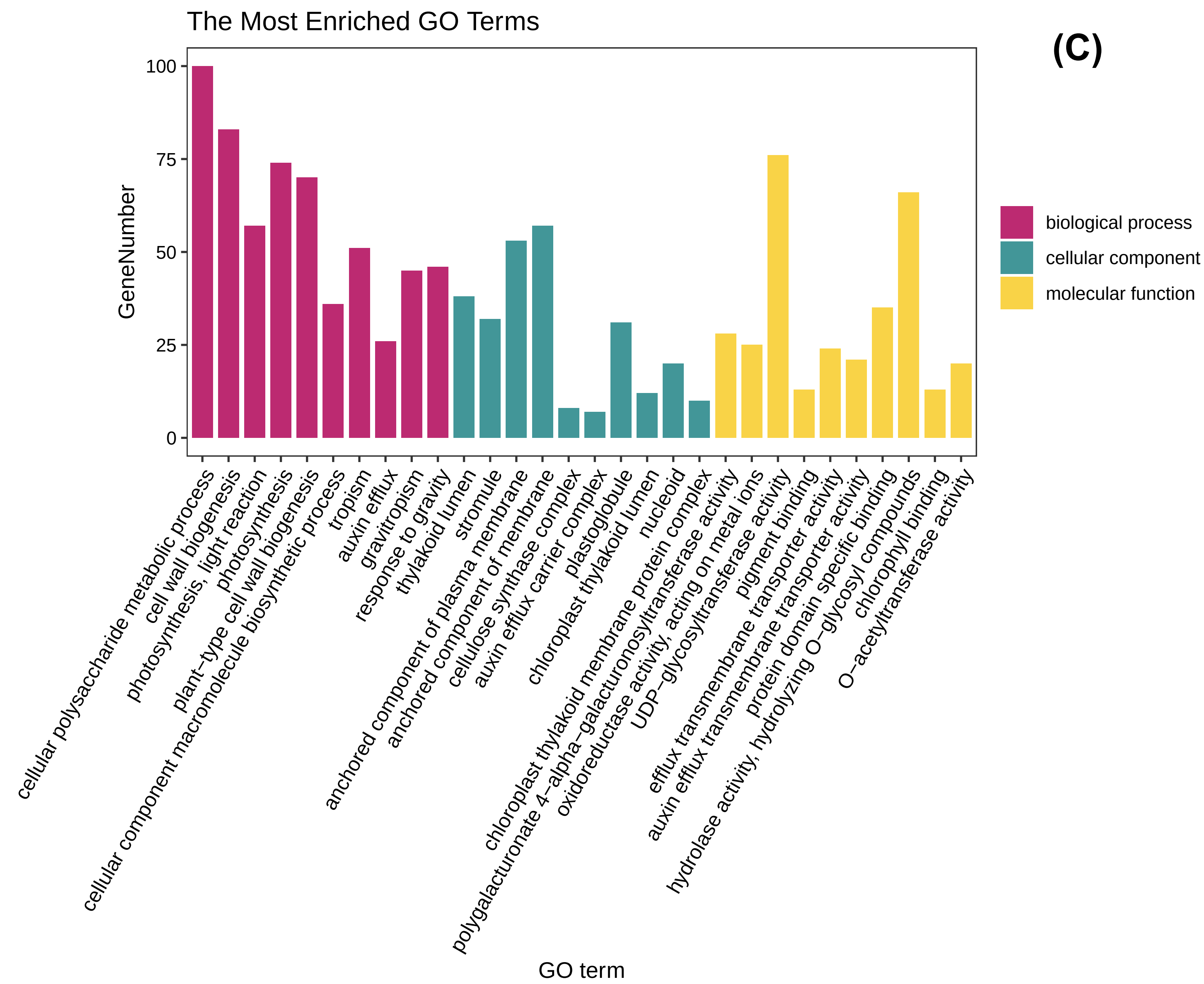
The Most Enriched GO Terms
(C)
GeneNumber
100
75
50
25
0
GO term
biological process
cellular component
molecular function
cellular polysaccharide metabolic process
cell wall biogenesis
photosynthesis, light reaction
photosynthesis
plant−type cell wall biogenesis
cellular component macromolecule biosynthetic process
tropism
auxin efflux
gravitropism
response to gravity
thylakoid lumen
stromule
anchored component of plasma membrane
anchored component of membrane
cellulose synthase complex
auxin efflux carrier complex
plastoglobule
chloroplast thylakoid lumen
nucleoid
chloroplast thylakoid membrane protein complex
polygalacturonate 4−alpha−galacturonosyltransferase activity
oxidoreductase activity, acting on metal ions
UDP−glycosyltransferase activity
pigment binding
efflux transmembrane transporter activity
auxin efflux transmembrane transporter activity
protein domain specific binding
hydrolase activity, hydrolyzing O−glycosyl compounds
chlorophyll binding
O−acetyltransferase activity

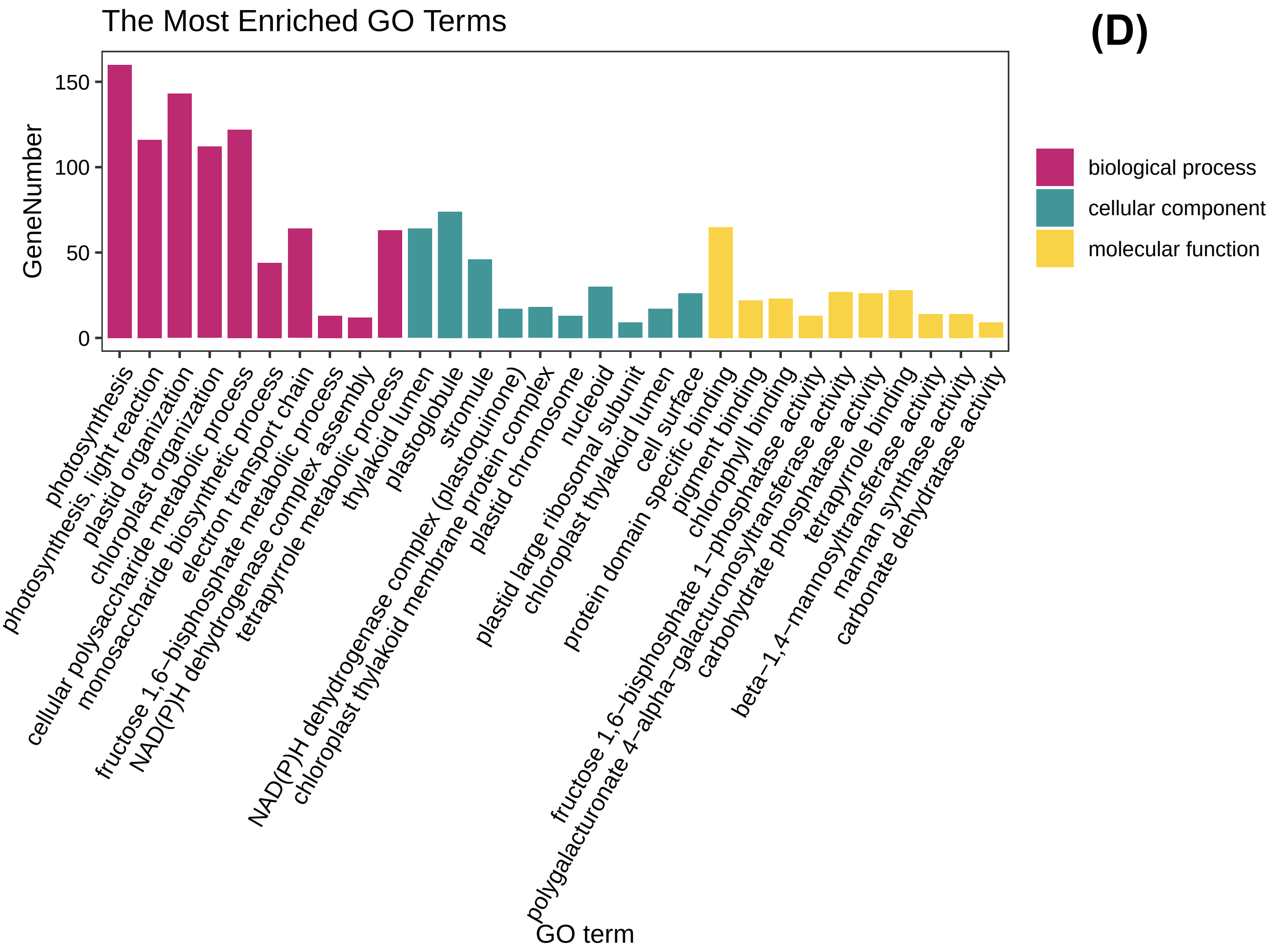

Figure S4. GO enrichment analysis results of different genes. Different colors represent different GO categories. Y-axis represents the number of genes in each function; X-axis displays the GO terms, and only the top 10 go terms of each category are displayed. (A)Up-regulated genes at 7 days of drought stress; (B)Up-regulated genes at 14 days of drought stress; (C) Down-regulated genes at 7 days of drought stress; (D) Down-regulated genes at 14 days of drought stress.
