# Supplementary material for: Combined GWAS and Transcriptome Analyses Provide New Insights Into the Response Mechanisms of Sunflower Against Drought Stress
Source: Front Plant Sci. 2022 May 3;13:847435. doi: 10.3389/fpls.2022.847435 (PMC9111542; doi:10.3389/fpls.2022.847435)
Supplement: Supplementary file 5 [file Data_Sheet_3.PDF]

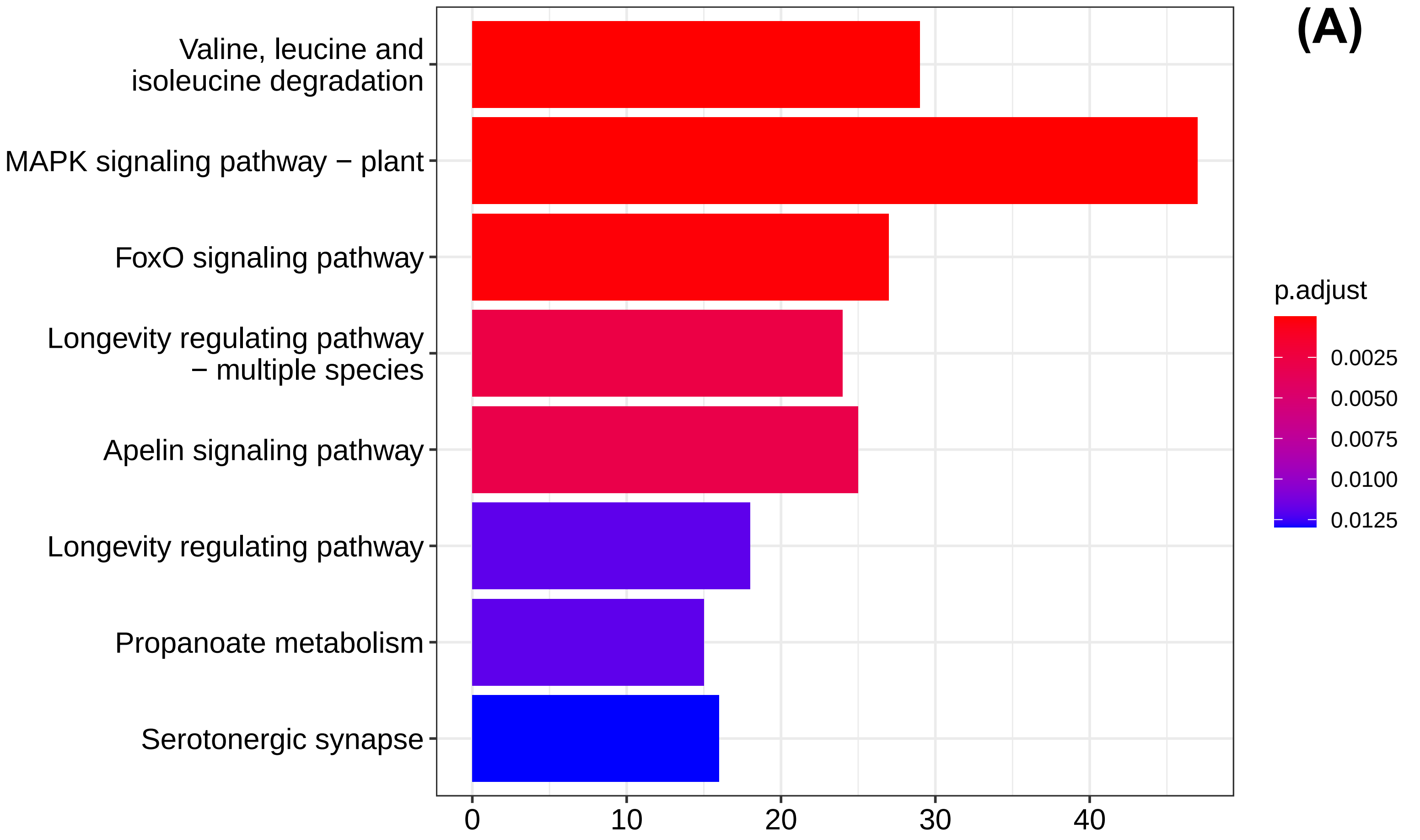
(A)
Valine, leucine and isoleucine degradation
MAPK signaling pathway − plant
FoxO signaling pathway
Longevity regulating pathway − multiple species
Apelin signaling pathway
Longevity regulating pathway
Propanoate metabolism
Serotonergic synapse
0
10
20
30
40
p.adjust
0.0025
0.0050
0.0075
0.0100
0.0125

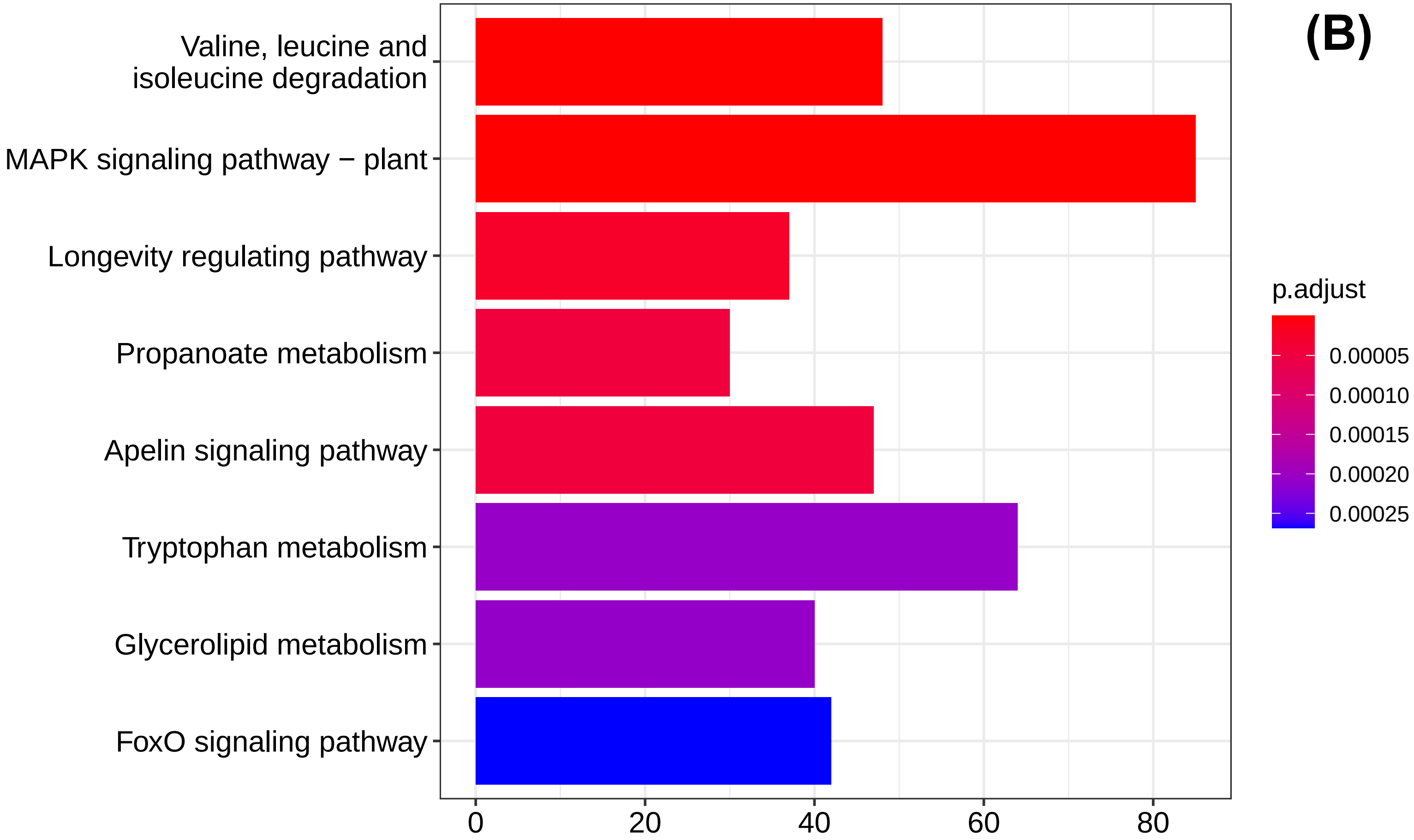
(B)
Valine, leucine and isoleucine degradation
MAPK signaling pathway − plant
Longevity regulating pathway
Propanoate metabolism
Apelin signaling pathway
Tryptophan metabolism
Glycerolipid metabolism
FoxO signaling pathway
0
20
40
60
80
p.adjust
0.00005
0.00010
0.00015
0.00020
0.00025

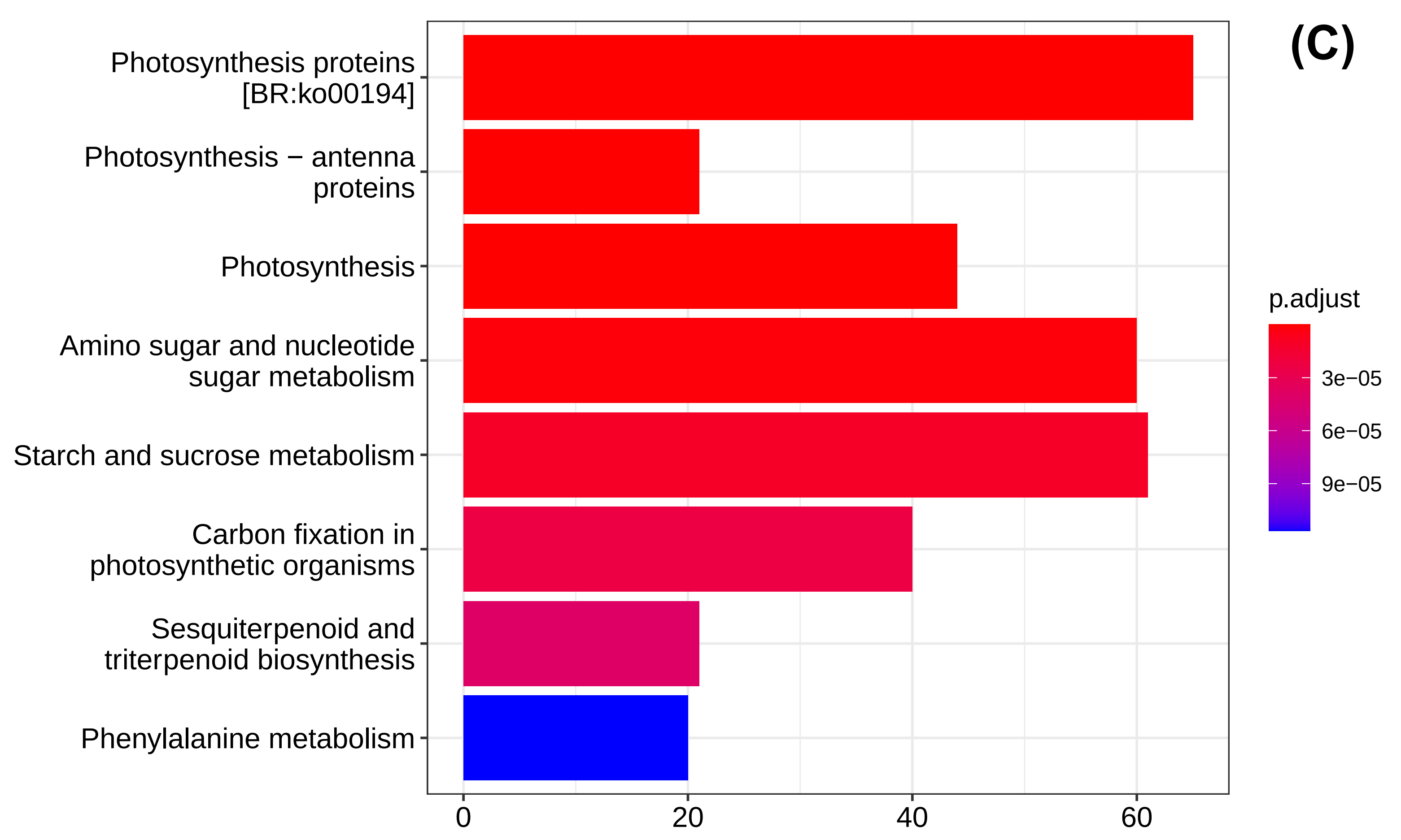
(C)
Photosynthesis proteins [BR:ko00194]
Photosynthesis − antenna proteins
Photosynthesis
Amino sugar and nucleotide sugar metabolism
Starch and sucrose metabolism
Carbon fixation in photosynthetic organisms
Sesquiterpenoid and triterpenoid biosynthesis
Phenylalanine metabolism
0
20
40
60
p.adjust
3e−05
6e−05
9e−05

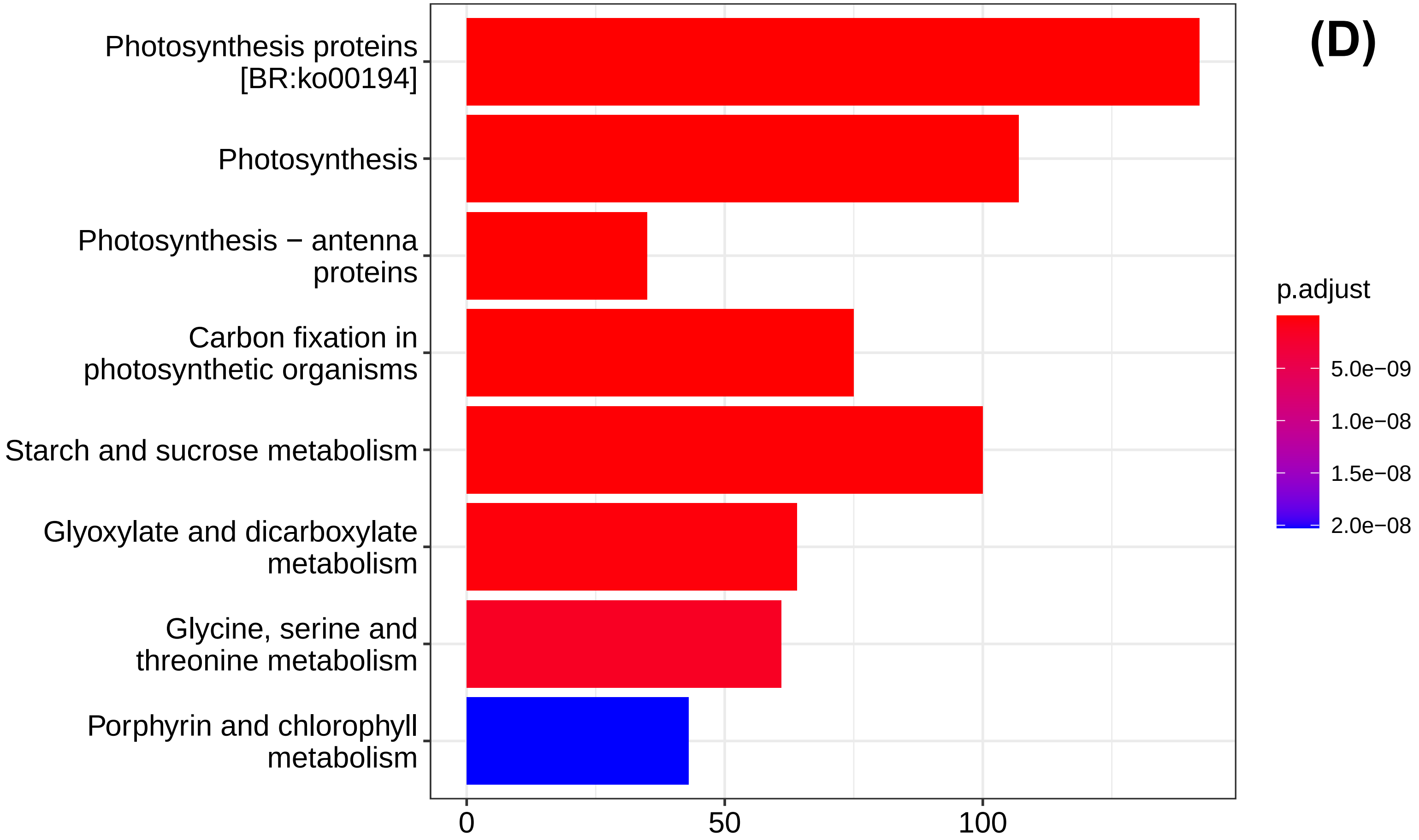

(D)
Photosynthesis proteins [BR:ko00194]
Photosynthesis
Photosynthesis − antenna proteins
Carbon fixation in photosynthetic organisms
Starch and sucrose metabolism
Glyoxylate and dicarboxylate metabolism
Glycine, serine and threonine metabolism
Porphyrin and chlorophyll metabolism
0
50
100
p.adjust
5.0e−09
1.0e−08
1.5e−08
2.0e−08

**Figure S5. KEGG enrichment of the DEGs in K58 under different drought stress time. The Y-axis represents the KEGG pathway. The X-axis represent the gene number enriched in each pathway. (A) Up-regulated genes at 7 days of drought stress. (B) Up-regulated genes at 14 days of drought stress. (C) Down-regulated genes at 7 days of drought stress. (D) Down-regulated genes at 14 days of drought stress.**
