# Supplementary material for: Combined GWAS and Transcriptome Analyses Provide New Insights Into the Response Mechanisms of Sunflower Against Drought Stress
Source: Front Plant Sci. 2022 May 3;13:847435. doi: 10.3389/fpls.2022.847435 (PMC9111542; doi:10.3389/fpls.2022.847435)
Supplement: Supplementary file 6 [file Data_Sheet_4.PDF]

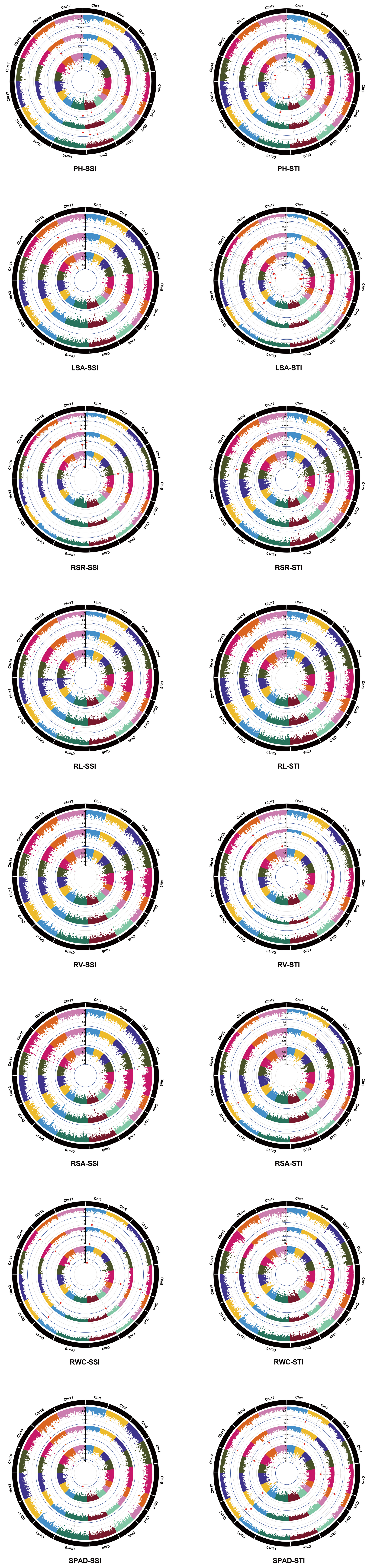

Figure S2. Circle Manhattan plots of genome-wide association mapping results. The three rings from outside to inside represent the results obtained by BLINK, FarmCPU and BLINK methods, respectively. Radial grey-dashed lines occur along the same vector as the associated significant marker, with the same location denoted across plots. The general significant trait-associated SNPs threshold are distinguished by the blue line. Significant markers above each threshold have been enlarged for clarity.
